# Molars and incisors: show your microarray IDs

**DOI:** 10.1186/1756-0500-6-113

**Published:** 2013-03-26

**Authors:** Virginie Laugel-Haushalter, Marie Paschaki, Christelle Thibault-Carpentier, Doulaye Dembelé, Pascal Dollé, Agnès Bloch-Zupan

**Affiliations:** 1 Developmental Biology and Stem Cells Department, Institut de Génétique et de Biologie Moléculaire et Cellulaire (IGBMC), Centre National de la Recherche Scientifique (UMR 7104), Institut National de la Santé et de la Recherche Médicale (U 964), Fédération de Médecine Translationnelle de Strasbourg (FMTS), Université de Strasbourg, BP 10142, 1 rue Laurent Fries, Illkirch Cedex, 67404, France; 2 Microarray and Sequencing Platform, Institut de Génétique et de Biologie Moléculaire et Cellulaire (IGBMC), Centre National de la Recherche Scientifique (UMR 7104), Institut National de la Santé et de la Recherche Médicale (U 964), Université de Strasbourg, Illkirch-Strasbourg, France; 3 University of Strasbourg, Faculty of Dentistry, 1 place de l'Hôpital, Strasbourg, France; 4 Reference Centre for Orodental Manifestations of Rare Diseases, Pôle de Médecine et Chirurgie Bucco-dentaires, Hôpitaux Universitaires de Strasbourg (HUS), Strasbourg, France

**Keywords:** Tooth development, Molar, Incisor, Gene expression, Mouse, Microarray

## Abstract

**Background:**

One of the key questions in developmental biology is how, from a relatively small number of conserved signaling pathways, is it possible to generate organs displaying a wide range of shapes, tissue organization, and function. The dentition and its distinct specific tooth types represent a valuable system to address the issues of differential molecular signatures. To identify such signatures, we performed a comparative transcriptomic analysis of developing murine lower incisors, mandibular molars and maxillary molars at the developmental cap stage (E14.5).

**Results:**

231 genes were identified as being differentially expressed between mandibular incisors and molars, with a fold change higher than 2 and a false discovery rate lower than 0.1, whereas only 96 genes were discovered as being differentially expressed between mandibular and maxillary molars. Numerous genes belonging to specific signaling pathways (the Hedgehog, Notch, Wnt, FGF, TGFβ/BMP, and retinoic acid pathways), and/or to the homeobox gene superfamily, were also uncovered when a less stringent fold change threshold was used. Differential expressions for 10 out of 12 (mandibular incisors versus molars) and 9 out of 10 selected genes were confirmed by quantitative reverse transcription-PCR (qRT-PCR). A bioinformatics tool (Ingenuity Pathway Analysis) used to analyze biological functions and pathways on the group of incisor versus molar differentially expressed genes revealed that 143 genes belonged to 9 networks with intermolecular connections. Networks with the highest significance scores were centered on the TNF/NFκB complex and the ERK1/2 kinases. Two networks ERK1/2 kinases and tretinoin were involved in differential molar morphogenesis.

**Conclusion:**

These data allowed us to build several regulatory networks that may distinguish incisor versus molar identity, and may be useful for further investigations of these tooth-specific ontogenetic programs. These programs may be dysregulated in transgenic animal models and related human diseases leading to dental anomalies.

## Background

A key question in developmental biology is how several shared molecular pathways can give rise to distinct organs, differing in their shape and tissue organization. The dentition represents a valuable system to address the issue of differential gene expression leading to the generation of specific tooth types. The mouse dentition is composed of one incisor and three molars on each hemiquadrant, separated by a toothless gap called diastema. Although molars and incisors develop according to the same basic developmental sequences, they display several important differences. Rodent incisors have a continuously growing ability through life, linked to the presence of an active stem cell niche located within the apical cervical loops [1]. They also exhibit asymmetrical development: ameloblasts differentiate and deposit enamel matrix only on the labial side, whereas the lingual side functions as a root analogue generating odontoblasts [2].

Odontogenesis proceeds through several stages. It initiates at the dental lamina stage by the appearance of a thickened area in the oral ectoderm, and proceeds to bud, cap and bell stages, odontoblasts and ameloblasts terminal differentiations, dentin and enamel matrix deposition and mineralization, root formation and finally tooth eruption. Odontogenesis is controlled by epithelio-mesenchymal interactions between neural crest-derived ectomesenchymal cells and oral ectoderm [3-9], and is regulated by conserved signaling pathways (FGF, BMP, Shh, Wnt, TGFβ, Notch, TNF/NFκB) [10-16]. Transcription factors including several homeobox gene products [17-21] and genes from the retinoic acid pathway [22] also play a role in tooth development.

The differential location, identity, shape and size of teeth are determined by several pathways acting at early stages of development [23]. At mouse embryonic day E10.5 the first molecular signals (BMP4, FGF8) initiating differential tooth morphogenesis are found in the oral ectoderm in mutually exclusive and complementary territories [9,24], which will trigger subsequent mesenchymal signaling. Already at this stage presumptive molar and incisor fields are well defined [25]. Tooth development was postulated not to involve any Hox (*Antennapedia*-like homeobox) gene [26], although recent studies showed specific expression of some Hox genes in distinct tooth bud tissues [27]. A number of other homeobox genes are expressed, however, in nested patterns across the developing jaws. The mandible is divided into oral (expressing *Lhx6* and *7*), aboral (expressing *Gsc*), distal (presumptive incisor, expressing *Msx1* and *2*) and proximal (presumptive molar, expressing *Dlx1* and *2*, *Barx1*, *Pitx1*) domains [17-19]. These expression patterns are defined by positive and negative signals from the oral epithelium. *Bmp4*, for example, is initially expressed in the distal epithelium and induces expression of *Msx1* in the underlying (presumptive incisor) mesenchyme, while at the same time it negatively regulates expression of *Barx1*, so as to restrict its expression to the presumptive molar region [18]. *Fgf8*, meanwhile, is expressed adjacent to *Bmp4* in the proximal oral epithelium and positively induces *Barx1* expression in the underlying presumptive molar epithelium [18]. Other genes display also a differential expression pattern [28,29].

Tooth shape specification from the dental lamina stage is contained within the ectomesenchyme. At the cap stage (E14.5 in the mouse) the condensing dental mesenchymal papilla controls the growth and folding of the inner dental epithelium. Mesenchymal signals induce within the enamel organ the formation of a signaling center called the primary enamel knot. It is a transitory structure of non proliferative cells, which produces several signaling molecules [30] and is essential to crown and cusps development and shape. The patterning role of the mesenchyme and dental papilla has also been addressed by heterologous recombination experiments from E13 to E16 between molar and incisor dental papilla and enamel organs, allowing the development of teeth of shape and type corresponding to the mesenchymal identity [31,32]. Many genes have a dynamic expression pattern during odontogenesis, and by the E14.5 cap stage a lot of genes that have earlier been linked to either the incisor or molar regions are expressed in all tooth germs and may not anymore be differentially expressed.

Alterations of these precisely regulated molecular and cellular sequences of development lead to dental anomalies, i.e. anomalies of teeth number, shape and size, of hard structures (enamel and dentin), of root formation and eruption. These malformations are observed in transgenic mouse models [33,34] mimicking human diseases and within the clinical phenotypes of syndromes or rare genetic diseases [35,36]. Indeed, at least 900 of the ~ 7000 known rare diseases or syndromes include oro-dental anomalies. In some syndromes only molars and canines are affected, like in oto-dental syndrome caused by deletions of the *FGF3* gene and characterized by grossly enlarged molar teeth (globodontia) [37]. In other syndromes, only incisors are affected like in KBG syndrome caused by mutation in *ANKRD11* and characterized by intellectual disability associated with short stature, facial dysmorphism and macrodontia of the upper central incisors, often with an agenesis of maxillary lateral incisors [38]. *SATB2* was involved in dental anomalies like incisor agenesis both in human in the 2q33.1 microdeletion syndrome [39] and in the corresponding mouse model [40]. We also recently identified *SMOC2*, a gene causing when mutated severe developmental dental defects with a dentin dysplasia phenotype associated to major microdontia, oligodontia, and shape abnormalities [41]. Furthermore, we showed a differential expression of this gene between molars and incisors.

In order to discover new candidate genes involved in the molecular events responsible for differential histomorphogenesis of the molars and incisors, we performed a transcriptomic analysis of developing murine lower incisors, mandibular molars and maxillary molars at the cap stage of development (E14.5). Here we report a global analysis of the identified differentially expressed genes. These data allowed us to build several regulatory networks that may distinguish incisor versus molar development, and may be useful for further investigations of these tooth-specific ontogenetic programs, some of which may be dysregulated in human diseases.

## Results and discussion

### Analysis of tooth specific transcriptional profiles

We decided to compare gene expression profiles in developing murine lower incisor and molars, as well as between the lower and upper (mandibular and maxillary) first molars. The developing tooth buds were collected by microdissection from E14.5 wild-type C57BL6 mice, and total RNA was extracted with the RNAeasy micro Kit (Qiagen, see Materials and Methods), after pooling 4 tooth germs per sample in order to obtain enough RNA for microarray hybridization. Altogether, 4 lower incisors samples, 4 maxillary molars samples, and 8 mandibular molars samples were hybridized on Affymetrix mouse gene 1.0 ST microarrays. Principal component analysis (PCA) was performed using the Partek Software to assess the consistency of the results. According to this analysis, the transcriptional profiles of three incisors samples (one sample of dubious quality was discarded) and eight mandibular molars samples showed that samples segregated in two distinct groups, showing relevant transcriptional differences between mandibular molars and lower incisors (Additional file 1). PCA performed on transcriptional profiles of eight mandibular molars samples and four maxillary molars samples also showed a clear segregation of samples between the two groups. This analysis indicated that transcriptional differences existed both between lower incisors and molars, as well as between mandibular and maxillary molars.

Our microarray data analysis allowed identification of several genes already known to be involved in tooth development (see Introduction), which did not show statistically different expression levels between distinct tooth samples. For instance, there was no significant difference in *Bmp4, Fgf8, Msx1*, *Pitx1, Pitx2, Gsc, Dlx2, Runx2, Msx2, Lhx6, Hand1* or *Satb2* expression between lower incisors and mandibular molars, and in *Bmp4, Fgf8, Msx1, Dlx2, Runx2, Msx2* and *Satb2* expression between mandibular and maxillary molars. Altogether, these data validated the sensitivity of the microarray analysis, and confirmed that several important regulators of tooth development were expressed at comparable levels in distinct tooth types at the stage analyzed.

**Figure 1:**
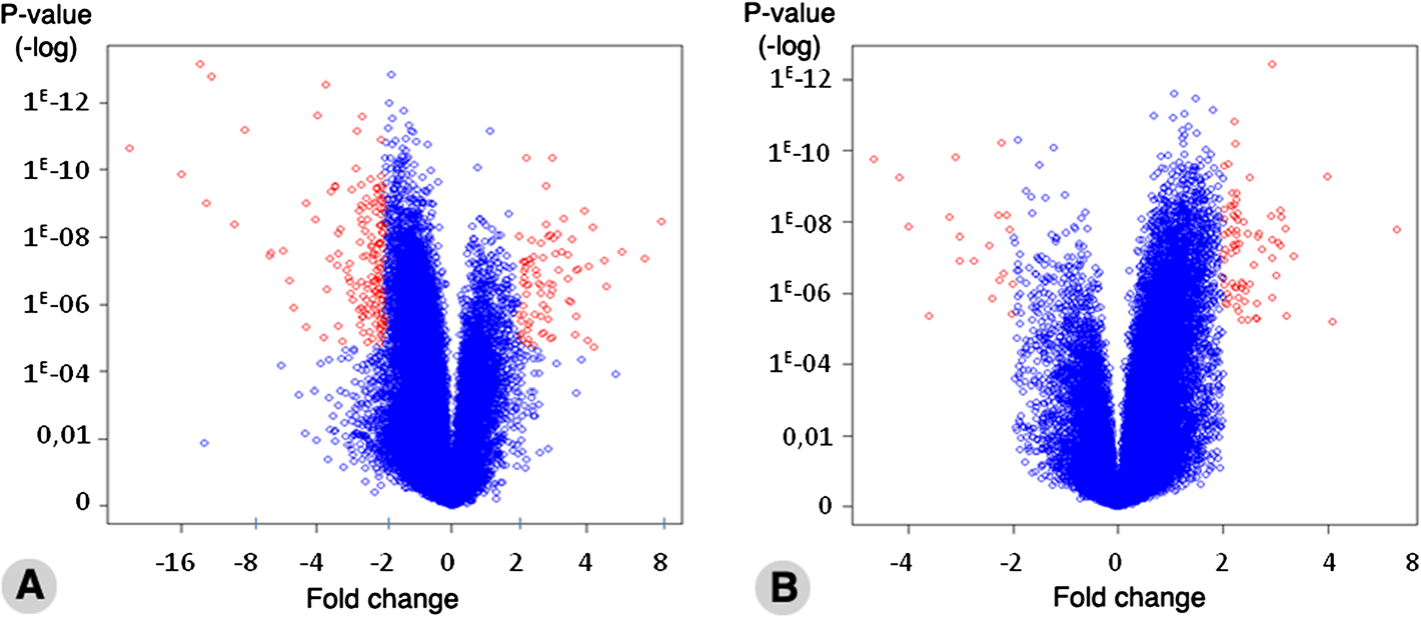
**Overview of gene expression changes in mandibular molars vs. incisors (A) and mandibular vs. maxillary molars (B). **Genes are plotted (Volcano plot) according to their fold change in mRNA expression (abscissae) and the corrected p-values from Student t-test (ordinates). In both plots, positive values correspond to genes more highly expressed in mandibular molars, and negative values to genes enriched in expression in incisors (A) or maxillary molars (B). Genes with a fold change in expression higher than 2 and a false discovery rate lower than 0.1 are shown in red.

Many genes exhibited statistically significant differential expression levels between specific tooth types. The distribution of differentially expressed genes is illustrated in Figure 1A (mandibular incisor versus first molar) and 1B (mandibular versus maxillary first molar). In these diagrams—as well as in all subsequent Tables—negative “fold changes” reflect an enriched expression in incisor (Figure 1A) or in maxillary molar (Figure 1B), whereas positive values indicate an enriched expression in mandibular molar. Genes plotted in red are those exhibiting a fold change superior to 2 (> 2 fold change) between the two types of samples (with statistical significance). We focused our analysis on such genes exhibiting at least a 2 fold change in expression in a given tooth type. However, in order not to overlook genes that may be relevant even if their differential expression is not as pronounced, we also considered all genes belonging to specific signaling pathways (the Hedgehog, Notch, Wnt, FGF, TGFβ/BMP, and retinoic acid pathways) and/or to the homeobox gene superfamily, as many important regulators of tooth development belong to these families. For analysis of these selected pathways and gene families, we applied a less stringent threshold (+ or - 1.2 fold change). For all identified genes we performed a detailed analysis of the literature, and hereby distinguish genes previously reported to be expressed (and sometimes functionally involved) in tooth development—including rare cases of reported differential expression between tooth types—from new candidate genes potentially involved in establishing tooth-specific programs.

### Expression profiling of mandibular molars versus incisors

#### Genes with a fold change higher than 2

Among the 35,556 probe sets represented in the microarrays, about 10% of the genes were excluded for any further analysis because of their low expression level, and 231 genes were differentially expressed between mandibular incisor and molar, with a fold change higher than 2 and a false discovery rate lower than 0.1 (corresponding to a p-value lower than 6.89E-04) (Figure 1A). The top ten genes exhibiting the highest expression in mandibular molars were *Barx1*, *C1qtnf3*, *Adcy8*, *Cntn6, Six2*, *Tcfap2b, Odz1, Vstm2a, Nptx1* and *Has2.* The top ten genes showing highest expression in incisors were *Hpse2*, *Alx1*, *Hand2*, *Sfrp4*, *Pax3*, *Alx3, Isl1, Mcpt2, Cacna2d3* and *Irx4* (Table 1A). Interestingly, among these genes, four were already known to be differentially expressed, with *Barx1* and *Six2* preferentially expressed in molars [42,43] and *Isl1* and *Hand2* in incisors [29,44].

**Table 1:** Overview of genes differentially expressed between lower (mandibular) incisor and molar

**A**	**Genes**	**Fold change**	**P-value**	**Known in teeth**	**Known as differentially expressed**	**References**
**Enriched in molars**	*Barx1*	8.60	3.47E-09	Yes	Yes	[42]
	*C1qtnf3*	7.30	4.37E-08	No	No	
	*Adcy8*	5.75	2.76E-08	No	No	
	*Cntn6*	4.91	2.94E-07	No	No	
	*Six2*	4.82	4.84E-08	Yes	Yes	[43]
	*Tcfap2b*	4.31	1.88E-05	No	No	
	*Odz1*	4.24	5.02E-09	No	No	
	*Vstm2a*	4.09	7.28E-08	No	No	
	*Nptx1*	4.06	1.21E-05	No	No	
	*Has2*	3.94	1.66E-09	No	No	
**Enriched in incisors**	*Irx4*	-5.31	1.92E-07	No	No	
	*Cacna2d3*	-5.66	2.55E-08	No	No	
	*Mcpt2*	-6.39	2.90E-08	No	No	
	*Isl1*	-6.44	3.53E-08	Yes	Yes	[29]
	*Alx3*	-8.36	6.59E-12	No	No	
	*Pax3*	-11.72	1.68E-13	Yes	No	[45]
	*Sfrp4*	-12.38	9.88E-10	No	No	
	*Hand2*	-13.20	6.89E-14	Yes	Yes	[44]
	*Alx1*	-15.95	1.35E-10	No	No	
	*Hpse2*	-27.41	2.23E-11	No	No	
**B**						
**Enriched in molars**	*Lhx6*	3.69	8.95E-08	Yes	Yes	[46]
	*Sfrp1*	3.56	7.94E-06	Yes	No	[47]
	*Smoc2*	3.28	3.28E-08	Yes	Yes	[41]
	*Shox2*	2.85	1.08E-08	Yes	No	[48]
	*Dlx1*	2.81	4.49E-11	Yes	Yes	[17]
	*Fgf12*	2.22	3.32E-06	Yes	No	[49]
	*Sfrp2*	2.16	4.40E-11	No	No	
	*Dbx2*	1.97	8.51E-06	Yes	No	[50]
	*Six4*	1.95	8.05E-07	Yes	Yes	[43]
	*Six1*	1.83	8.69E-06	Yes	Yes	[43]
	*Lhx8*	1.64	2.87E-06	Yes	No	[51]
	*Bmpr1a*	1.36	7.12E-07	Yes	No	[52]
	*Mapk1*	1.30	3.20E-06	Yes	No	[53]
**Enriched in incisors**	*Gas1*	-1.25	6.93E-06	Yes	No	[54]
	*Hoxa2*	-1.41	9.43E-07	Yes	No	[26]
	*Tlx2*	-1.47	7 .35E-07	No	No	
	*Irx6*	-1.58	3.09E-08	No	No	
	*Gdf6*	-2.00	4.01E-06	Yes	No	[55]
	*Aqp1*	-2.16	9.51E-08	Yes	No	[56]
	*Amtn*	-2.28	1.65E-08	Yes	Yes	[57]
	*Prtg*	-2.3	9.93E-07	Yes	No	[58]
	*Slitrk6*	-2.37	2.60E-07	Yes	No	[59]
	*Wnt5a*	-2.38	1.38E-05	Yes	No	[60]
	*Hand1*	-2.55	4.01E-06	Yes	Yes	[28]
	*Prkcq*	-2.75	7.17E-07	Yes	No	[61]
	*Tlx1*	-2.84	6.05E-08	Yes	No	[62]
	*Bmp5*	-3.20	3.00E-08	No	No	
	*Cyp26c1*	-4.05	3.10E-09	Yes	No	[63]
	*Nts*	-4.43	9.56E-10	Yes	No	[64]
	*Irx4*	-5.31	1.92E-07	No	No	

The table is subdivided in two sections highlighting: (A) The "top ten" genes showing the highest degree of enrichment in incisor (negative values) or molar (positive values); (B) additional examples of differentially expressed genes, some already known from the literature as being expressed in developing teeth, others not predicted from the literature. Separate columns indicate those genes already known as being expressed in developing teeth ("Known in teeth"), and sometimes as being differentially expressed in both tooth types ("Known as differentially expressed"). In all cases, one relevant reference has been selected. For a complete list of differentially expressed genes (with fold changes > 2 or < -2), see Additional file 2.

Additional literature searches revealed that, among the remaining 231 genes, only 22 were previously described as being expressed during tooth development, including 9 genes known to be differentially expressed between molar and incisors. Thus, our analysis revealed nearly 200 “new”, potentially interesting genes not previously described as differentially expressed between developing incisors and molars (a complete list is given in Additional file 2). Table 1B provides data for selected genes with high fold change and/or belonging to families for which other member(s) are involved in odontogenesis. *Sfrp2* and *Sfrp4*, for example, belong to the family of secreted frizzled-related proteins, for which *Sfpr1* was already known to be expressed in teeth [65,66]. *Tlx2* and *Bmp5* also have two paralogues, *Tlx1* and *Bmp4,* that were previously described as being differentially expressed between tooth types [62,67]. Two *Alx* genes, *Alx1* and *Alx3* were differentially expressed in our microarray experiments, with very high fold changes. The corresponding human genes are mutated in frontonsal dysplasia affecting the midline facial structures [68]. We also found two members of the *Iroquois* homeobox gene family, *Irx4* and *Irx6*, suggesting a role of these genes in defining incisor identity.

#### Genes from selected pathways or families

From the 3078 genes exhibiting a fold change higher than 1.2 and a false discovery rate lower than 0.1, 107 belonged to pathways or families selected as being important for tooth development (the FGF, TGFβ/BMP, Wnt, Hedgehog, retinoic acid, and Notch pathways, and the homeobox gene superfamily: see Introduction). Among these, 88 had not been reported to be expressed in teeth and were considered as new potential genes involved in tooth development (Table 2). Nineteen genes were already known to be expressed in teeth (Table 2, gene names in bold), and among them 11 were known to be differentially expressed between the two tooth types (Table 2, underlined). Considering genes not previously known to be expressed in teeth, and genes not yet described to be differentially expressed between tooth types, we found in total 99 genes not yet involved in differential tooth morphogenesis.

**Table 2:** Overview of genes belonging to selected signaling pathways (FGF, TGFβ/BMP, Wnt, Hedgehog, Retinoic acid, Notch) or to the homeobox-containing superfamily, showing differential expression in mandibular molar or incisor

**Pathway/Family**	**Gene names (fold change)**	**Number of genes**	**Differentially expressed in teeth**	**Not known to be differentially expressed in teeth**	**New genes in teeth**	**Total new genes and new differentially expressed genes**
FGF: molars	***Fgf12 (2.24)***	1	0	1	0	1
FGF: incisors	*Fgf22 (-1.41)*	1	0	0	1	1
TGFβ: molars	*Thbs2 (1.77); Gdf7 (1.73); ****Bmpr1a (1.36);****Ppp2r1b (1.35); ****Mapk1 (1.3);****Smurf2 (1.25)*	6	0	2	4	6
TGFβ: incisors	***Bmp5 (-3.20);****Gdf6 (-2.00); Acvr1c (-1.59); Inhbe (-1.52); Nodal (-1.45)*	5	0	1	4	5
Wnt: molars	***Sfrp1 (3.56);****Sfrp2 (2.16); Plcb4 (1.66); Camk2d (1.48); Ccnd2 (1.44); Ppp2r1b (1.35); Cul1 (1.30); Ppp3cb*	8	0	1	7	8
Wnt: incisors	*Sfrp4 (-12.38); ****Wnt 5a (-2.38);****Cer1 (-1.64); Wnt9b (-1.51); Wnt1 (-1.45); Camk2a(-1.34); Ppp3r2 (-1.32)*	7	0	1	6	7
Hedgehog incisors	*Ihh (-1.30); Btrc (1.26); ****Gas1 -(1.25)***	3	0	1	2	3
Retinoic acid: molars	***Cyp1b1;****Nr2f1 (3.59); Nr2f2 (2.69). Aldh7a1 (1.50)*	4	0	0	4	4
Retinoic acid: incisors	***Cyp26c1 (-4.05);****Cyp2c54 (-2.26); Rdh1 (-1.94); Cyp2c66 (-1.81); Cyp2a12 (-1.76); Rarres1 (-1.57); Rdh9 (-1.57); Ugt1a9 (-1.57); Aldh1b1 (-1.51); Rbp3 (-1.49); Pram1 (-1.43); Rarres2 (-1.35); Adh7 (-1.33); Cyp2b19 (-1.31); Rdh8 (-1.29)*	15	0	1	14	15
Notch: incisors	*Rbpjkl (-2.12); predicted gene 5109 (-1.71); Dll1 (-1.37)*	3	0	0	3	3
Homeobox genes: molars	***Barx1 (8.59); Six2 (4.81); Lhx6 (3.69); Shox2****(2.85); ****Dlx1 (2.81); Dbx2 (1.97); Six4 (1.95); Six1 (1.83);Lhx8 (1.64)***	9	6	2	1	3
Homeobox genes: incisors	*Alx1 (-15.94 ) Alx3 (-8.36); ****Isl1 (-6.44 )****; Irx4 (-5.31); ****Tlx1 (-2.83);****Otx1 (-2.48) ; Hoxa11 (-1.92); Hoxd8 (-1.86); Hoxd3 (-1.84); Hoxd4 (-1.81); Obox5 (-1.76); Lbx2 (-1.74); Rhox6 (-1.72); Hoxd10 (-1.66); Rhox1 (-1.62); Lmx1b (-1.61); Hoxd11 (-1.60); Hoxd1 (-1.60); Nkx2-1 (-1.60);Hnf1b (-1.59); Hoxc6 (-1.58); Irx6 (-1.58); Sebox (-1.57); Hhex (-1.55); Lhx4 (-1.54); Rhox12 (-1.54); Rhox2a (-1.53); Hoxc4 (-1.52); Tlx2 (-1.47); Rhox7 (-1.46); Hoxa9 (-1.44); ****Hoxa2 (-1.41);****Pdx1 (-1.40); Hoxb9 (-1.40); Esx1 (-1.39); Crx (-1.36) ; Hoxb7 (-1.36); Hoxa6 (-1.35); Arx (-1.33); Dux (-1.33); Lbxcor1 (-1.32); Gsx2 (-1.29); Hmx2 (-1.27); Hoxb2 (-1.27); Gbx1 (-1.26)*	45	2	1	42	43
Total		**107**	**8**	**11**	88	**99**

Genes are listed as being enriched in expression in molar or incisor, with fold changes in expression in parentheses. Genes already known from the literature to be expressed in teeth appear in bold, and those for which a differential expression was reported for the two tooth types are underlined. Additional columns summarize the literature survey, scoring genes previously described as expressed in developing teeth ("Known in teeth"), as differentially expressed in both tooth types ("Known as differentially expressed"), or "new" (i.e. not described in the literature: right-most column).

#### Quantitative RT-PCR analysis

**Figure 2:**
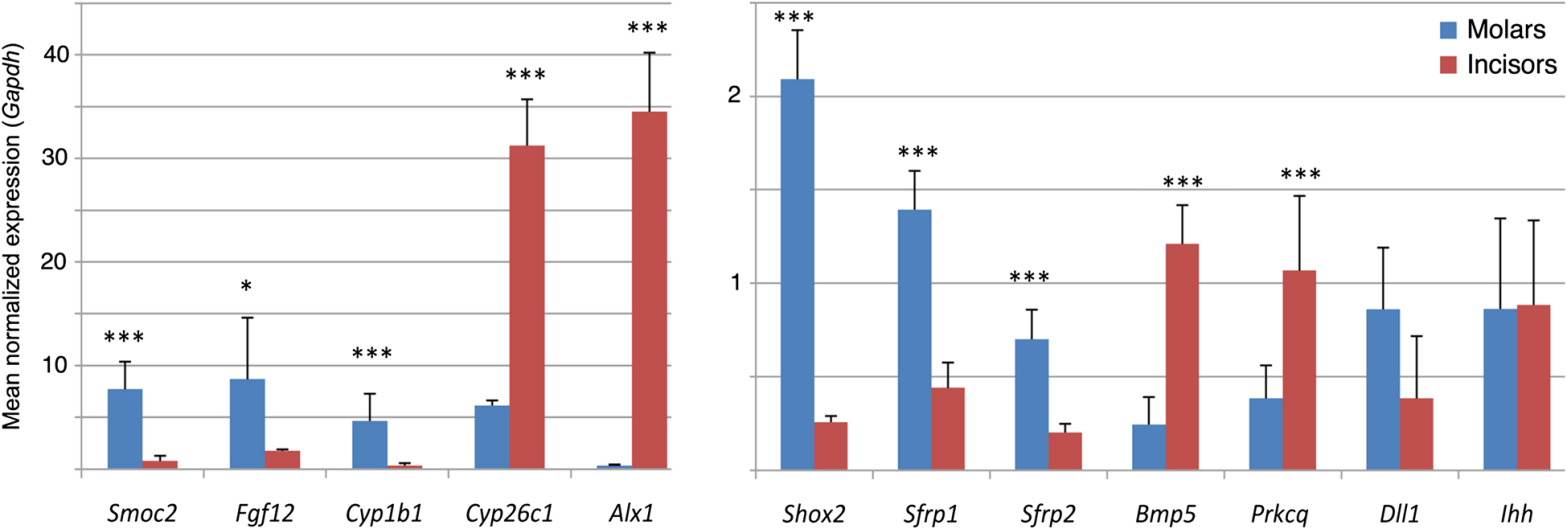
**Real-time quantitative RT-PCR analysis of genes selected for their differential expression between mandibular molars vs. lower incisors as detected by Affymetrix microarrays. **Histograms show expression levels in molars (blue) and incisors (red) as values normalized with respect to *Gapdh *expression. Data (mean ±SEM) were analyzed with Student t-test; ***p<0.001; **p<0.01; *p<0.05.

Twelve of these 99 genes were selected for validation of the microarray data by quantitative RT-PCR (qRT-PCR). These were candidates from interesting signaling pathways: *Ihh* (from the hedgehog pathway), *Dll1* (from the Notch pathway), *Sfrp1* and *Sfrp2* (from the Wnt pathway), *Fgf12* (from the FGF pathway), *Bmp5* (from the TGFβ pathway/superfamily), *Cyp26c1* and *Cyp1b1* (encoding two retinoic acid-metabolizing enzymes), *Alx1* and *Shox2* (members of the homeobox gene superfamily). We also decided to verify two genes known to be expressed in teeth and exhibiting a fold change higher than 2: *Smoc2* because we recently detected by in situ hybridization a differential expression between molars and incisors [41] and *Prkcq,* which belongs to the NFκB pathway (Table 1B). qRT-PCR was performed on RNA samples distinct from those used for microarray hybridization. From these 12 genes, 10 were found to be differentially expressed between molar and incisors by qRT-PCR, in agreement with the microarray data (Figure 2). The two exceptions were *Ihh*, which did not exhibit differential expression, and *Dll1*, which displayed an opposite expression (molar > incisor, not statistically significant) when compared to the microarray data.

#### Gene network analysis

**Figure 3:**
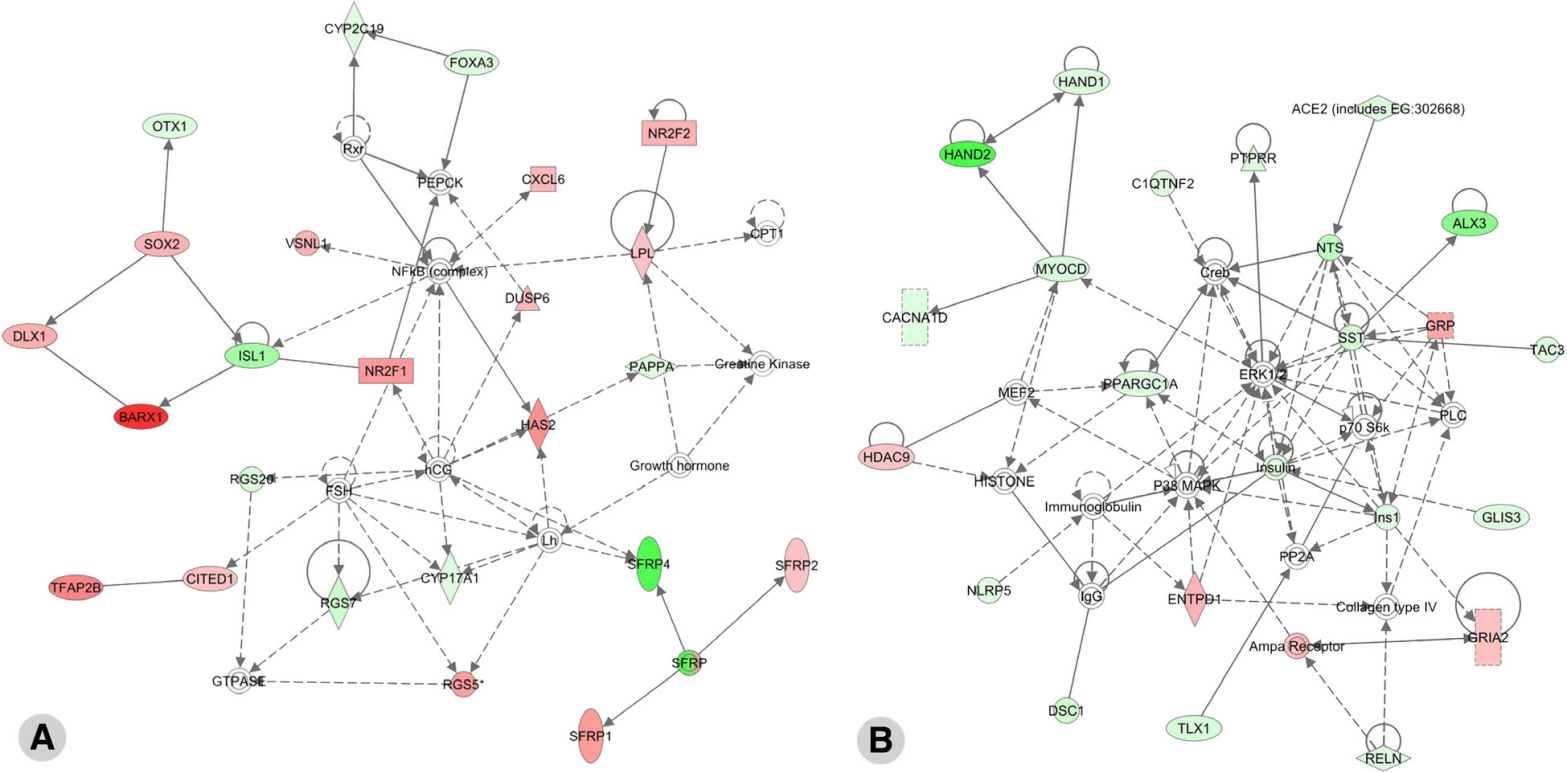
**Ingenuity pathway gene network analysis of incisor vs. molar expressed genes.** The two most significant gene networks identified in the Ingenuity pathway analysis of our microarray data are shown. These networks (see Results for details) are centered on the NFκB complex (**A**) and the ERK1/2 kinases (**B**). Many of the key genes highlighted in these networks are members of the ontology groups that include receptors, ligands and interacting proteins, and two families of transcription factors: homeodomain (homeobox encoded) proteins and nuclear receptors. The networks are displayed graphically as nodes (genes/gene products) and edges (biological relationships between the nodes). Differentially expressed genes are shown in two colors, the intensity of the colors reflecting the degree of enrichment in molar (red) versus incisor (green) tooth buds. Nodes are displayed using various shapes representing the functional class of the gene product (flat oval: transcription factor; tall oval: transmembrane receptor or interacting protein; losange: enzyme; triangle: kinase; rectangle: G protein-coupled receptor; circle: other). Interactions are depicted by arrows ("acts on", with dashed arrows indicating "indirect" interactions) or straight lines (binding only).

**Relevant networks when considering all genes with a fold change higher than 2 **To gain insight into interactions that may occur between the differentially expressed genes and/or proteins, we constructed biologically relevant networks using the Ingenuity pathway analysis software. From the 231 differentially expressed genes with a fold change higher than 2, 143 genes were mapped in nine networks. The most relevant network (score=48) was centered on the NFκB complex and contained 24 differentially expressed genes (Figure 3A). *Barx1, Dlx1, Sox2, Cited1, Nr2f1, Nr2f2, Vsnl1, Cxcl6, Dusp6, Has2, Lpl, Tfap2b, Rgs5, Sfrp1* and *Sfrp2* were more strongly expressed in mandibular molars than in incisors. *Otx1, Isl1, Cyp2c19, Foxa3, Pappa, Rgs7, Rgs20, Cyp17a1* and *Sfrp4* were more expressed in incisors. This network highlighted two genes from the nuclear receptor superfamily (*Nr2f1* and *Nr2f2*, also known as COUP-TFI and II), both expressed at higher levels in molars. On the other hand, several genes from the *Sfrp* family were differentially expressed, with *Sfrp1* and *Sfrp2* being more expressed in mandibular molars and *Sfrp4* more expressed in incisors (Figure 3A). The second network (score=42) was centered on ERK1/2 and contained 22 differentially expressed genes (Figure 3B). *Hdac9, Entpd1, Ampa Receptor, Grp and Gria2* were expressed at higher levels in mandibular molars, whereas *Hand1, Hand2, Myocd, Cacna1d, Ppargc1a, C1qtnf2, Ptprr, Ace2, Nts, SSt, Alx3, Ins1, Glis3, Nlrp5, Dsc1, Tlx1* and *Reln* were preferentially expressed in lower incisors. *Hand1* and *Hand2* were also preferentially expressed in the incisor area, as previously reported at E10.5 [44].

**Relevant networks when considering genes from selected pathways or families **From the 107 differentially expressed genes in the selected pathways, 50 genes were mapped in only 4 different networks involved in embryonic or tissue development (Additional file 3). The first network (score=24) contained 16 differentially expressed genes and was focused on *Six1,* a gene highly expressed in molars. This network contained two additional *Six* genes, *Six2* and *Six4,* also more expressed in molars, as well as *Six5,* which was not differentially expressed. It further contained *Irx4* and *6,* preferentially expressed in incisors, whereas *Irx2* was not differentially expressed. In this network we also found *Dlx1,* a gene preferentially expressed in molars, whereas *Dlx2* and *Dlx5* were not differentially expressed. *Hmx2, Arx, Gsx2, Crx* and *Wnt11* were more expressed in incisors. Some Hox genes appeared in this network. Hox genes were classically considered as being not expressed in the maxillo-mandibular region, which derives from the first embryonic branchial arch [26], but recent expression studies have revealed expression of some Hox genes in specific developing tooth compartments [27]. This network also contained genes known to act during tooth development, like *Bmp4* or *Fgf10,* but these were not differentially expressed.

The second network (score=20) contained 14 differentially expressed genes and was centered on *Shox2,* a homeobox gene known to be expressed in teeth at E14.5 (Additional file 3). Only this gene and *Sfrp2* were more expressed in molars in this network. All others genes like *Tlx2, Brtc, Cer1, Hhex*, or *Lmx1b,* were preferentially expressed in incisors. This nework also contained genes known to be involved in odontogenesis, like *Runx2* and *Pitx2*, and which were not differentially expressed. The third network was centered on *Pparg* (encoding PPARγ, a member of the nuclear receptor superfamily) and all molecules from this network were more highly expressed in incisors except one nuclear receptor gene, *Nr2f2* (encoding COUP-TFII). The fourth network was centered on *fos* and contained several genes from the retinoic acid pathway preferentially expressed in incisors (Additional file 3).

### Expression profiling of lower versus upper molars

#### Genes with a fold change higher than 2

We found 96 genes differentially expressed between mandibular and maxillary molars with a fold change higher than 2 (Table 3; Additional file 4 for a full list). The gene with highest expression in maxillary molars was *Cyp26c1*, a gene previously shown to be expressed in teeth [63] and found to be also differentially expressed between molars and incisors in our microarray analysis. The *Nefl* gene, responsible for Charcot Marie Tooth disease, was the most highly enriched in mandibular molars (Table 3A). *Nkx2-3* had already been reported in the literature as differentially expressed between the two tooth types [69] (Table 3A). Examination of *Nkx2-3* null mice revealed defects in maturation and cellular organization of the sublingual glands. Furthermore, cusps were absent from mandibular molars and the third molar was occasionally missing [69].

**Table 3:** Overview of genes differentially expressed between lower (mandibular) and upper (maxillary) molars

**A**	**Genes**	**Fold change**	**P-value**	**Known in teeth**	**Known as differentially expressed**	**References**
**Enriched in lower molars**	*Nefl*	6.28	1.68E-08	No	No	
	*Ostn*	4.12	6.22E-06	No	No	
	*Nkx2-3*	3.97	5.32E-10	Yes	Yes	[69]
	*Tnnt1*	3.19	9.08E-08	No	No	
	*Chrna1*	3.04	4.47E-06	No	No	
	*Nefm*	3.01	1.54E-08	No	No	
	*Myf5*	2.94	7.14E-09	No	No	
	*Klhl31*	2.92	4.58E-08	No	No	
	*Plac8*	2.91	4.68E-09	No	No	
	*Synpo2l*	2.87	3.71E-08	No	No	
**Enriched in upper molars**	*Naalad2*	-2.21	6.48E-09	No	No	
	*Kcnb2*	-2.29	1.42E-06	No	No	
	*Itga8*	-2.34	4.57E-08	No	No	
	*Atp6*	-2.84	1.26E-07	No	No	
	*Alx1*	-2.85	2.50E-08	No	No	
	*Gabrb2*	-3.06	7.32E-09	No	No	
	*Ndst4*	-3.48	4.21E-06	No	No	
	*Pla2g7*	-3.99	1.34E-08	No	No	
	*Nmbr*	-4.25	5.52E-10	No	No	
	*Cyp26c1*	-5.04	1.74E-10	Yes	No	[63]
**B**						
**Enriched in lower molars**	*Dlx6*	2.76	1.01E-07	Yes	No	[70]
	*Gsc*	2.21	1.31E-06	Yes	No	[71]
	*Pitx1*	2.19	6.09E-08	Yes	Yes	[19]
	*Prkcq*	2.15	6.71E-07	Yes	No	[61]
	*Barx2*	2.00	3.47E-07	Yes	No	[72]
	*Lhx9*	1.86	1.12E-06	No	No	
	*Nkx6-1*	1.62	4.43E-08	No	No	
	*Lhx1*	1.48	2.87E-06	No	No	
	*Msx3*	1.43	8.33E-07	No	No	
	*Nkx2-1*	1.40	1.84E-06	No	No	
**Enriched in upper molars**	*Gli1*	-1.34	6.21E-07	Yes	No	[73]
	*Dlx1*	-1.36	2.18E-06	Yes	No	[71]
	*Lhx8*	-1.48	2.57E-06	Yes	No	[51]

As for Table 1, this table is organized in two sections showing: (A) The top ten genes showing highest expression in mandibular (lower) molars (positive values) or maxillary (upper) molars (negative values); (B) examples of genes known from the literature as being expressed in developing teeth, only a minority being described as differentially expressed in upper vs. lower molars ("Known as differentially expressed"), or not described in the literature. Among the top ten genes, only two were known to be expressed in developing teeth. For a complete list of differentially expressed genes (with fold changes > 2 or < -2), see Additional file 4.

Other genes previously described as acting during odontogenesis were identified as being differentially expressed in our microarray analysis. Seven of them were expressed with a fold change higher than 2 (Table 3B). Among these, *Pitx1* had already been described as being differentially expressed between upper and lower molars [19]. Inactivation of the *Pitx1* gene in mice affected mandibular tooth morphogenesis [19].

Among the “new” genes unravelled by our microarray analysis, several belong to gene families with other members known to act during odontogenesis. *Lhx1* and *Lhx9* were identified as displaying enriched expression in mandibular molars (Table 3B). Their paralogues *Lhx6* and *Lhx7* are implicated in tooth patterning at E10.5 [46]. *Lhx6/7* double mutant embryos lacked molar teeth. Despite molar agenesis, *Lhx6/7*-deficient animals had normal incisors which, in the maxilla, were flanked by a supernumerary pair of incisor-like teeth [74]. *Nkx6-1* and *Nkx2-1* appeared interesting as their paralogue *Nkx2-3* is already known to be differentially expressed between mandibular and maxillary molars [69]. *Msx3* was identified as being enriched in mandibular molars; its homologues *Msx1* and *Msx2* are known to play a role in mouse dentition patterning at E10.5 [75]. *Alx1*, which was differentially expressed in mandibular molars vs. incisors in our microarray analysis, was also found to be expressed at higher levels in maxillary molars (Table 3A).

#### Genes from selected pathways or families

Among the 2070 genes with a fold change higher than 1.2 and a p-value lower than 0.1 in mandibular vs. maxillary molars, 61 belonged to the pathways or families selected for further analysis (Table 4). Only nine genes were known to be expressed in teeth (Table 4, in bold), four of these being reported to be differentially expressed betwen the two tooth types (Table 4, underlined). Fifty-three genes had not yet been described as being expressed or acting during odontogenesis. In total we found 58 new genes not known to be differentially expressed between the two tooth types.

**Table 4:** Overview of genes belonging to selected signaling pathways (FGF, TGFβ/BMP, Wnt, Hedgehog, Retinoic acid, Notch) or to the homeobox gene superfamily, showing differential expression in mandibular (inferior) versus maxillary (superior) molars

**Pathway/Family**	**Gene names (fold change)**	**Number of genes**	**Differentially expressed in teeth**	**Not known to be differentially expressed in teeth**	**New genes in teeth**	**Total new genes and new differentially expressed genes**
FGF: lower molars	Fgfr4 (2.58); *Fgf16 (1.86)*	2	0	0	2	2
TGFβ: lower molars	*Amhr2 (1.68); Acvr2b (1.26)*	2	0	1	1	2
TGFβ: upper molars	*Smad9 (-1.53)*	1	0	0	1	1
Wnt: lower molars	*Camk2a (1.83); Fzd8 (1.67); Wnt9b (1.55); Camk2b (1.50); Wnt11 (1.39); Plcb2(1.39); Fzd5 (1.29); Cer1 (1.26)*	8	0	0	8	8
Wnt: upper molars	*Vangl1 (-1.22)*	1	0	0	1	1
Hedgehog: upper molars	***Gli1 (-1.34)***	1	1	0	0	0
Retinoic acid: lower molars	*Rorb (2.09); Rbp2 (1.59); Crabp2 (1.58); Polr2l (1.37);Rdh8 (1.27)*	5	0	0	5	5
Retinoic acid: upper molars	***Cyp26c1 (-5.04);****Aldh1a1 (-1.98); Dhrs3 (-1.51)*	3	0	1	2	3
Notch: lower molars	*Dtx4 (1.81); Dll1 (1.41); Rfng (1.31); Dll3 (1.36)*	4	0	0	4	4
Homeobox genes: lower molars	***Nkx2-3 (3.97).Dlx6 (2.76); Pitx1 (2.18) ;Gsc (2.21); Barx2 (2.01);****Hoxa7 (1.91); Lhx9 (1.86); Rhox11 (1.82); Hoxb7 (1.78); Vsx1 (1.69); Phox2b (1.68); Hoxa6 (1.67); Rhox4f (1.65); Nkx6-1 (1.62); Hoxb9 (1.57); Hoxa10 (1.55); Tgif2 (1.53); Lhx1 (1.48); Hmx1 (1.47); Hoxa3 (1.47); Hoxb2 (1.46); Hoxc12 (1.43); Prox2 (1.43); Msx3 (1.43); Gsx1 (1.42); Mixl1 (1.40); Nkx2-1 (1.40); Hoxd8 (1.39); Gbx1 (1.38); Hmx2 (1.36); Lmx1a (1.34); Rhoxa2 (1.23)*	32	3	2	27	29
Homeobox genes: upper molars	*Alx1 (-2.85);****Lhx8 (-1.48); Dlx1 (-1.36)***	3	1	1	1	2
Total		**62**	**4**	**5**	53	**58**

Fold changes in expression are indicated in parentheses. As in Table 2, genes known from the literature to be expressed in teeth appear in bold, and those for which a differential expression was reported are underlined. Additional columns scoring the genes previously described as being expressed in developing teeth ("Known in teeth"), as being differentially expressed in both molar types ("Known as differentially expressed"), or "new" (not previously described).

#### Quantitative RT-PCR analysis

**Figure 4:**
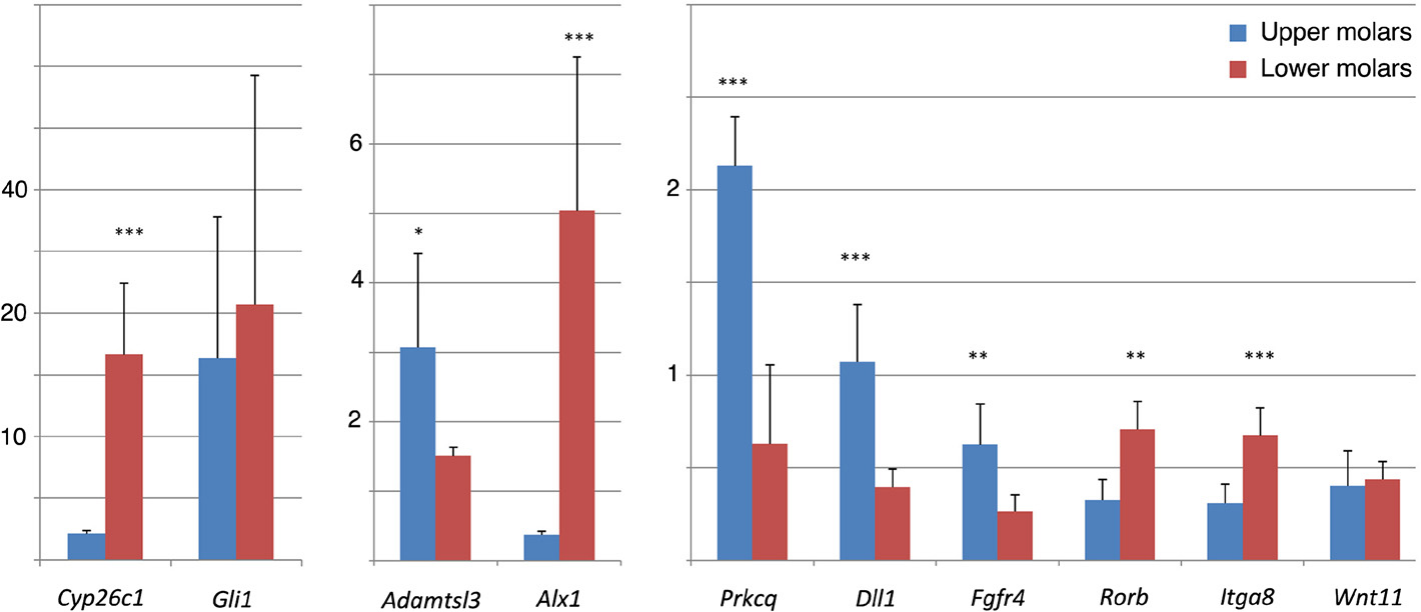
**Real-time quantitative RT-PCR analysis of genes selected for their differential expression between mandibular vs. maxillary molars as detected by Affymetrix microarrays. **Histograms show expression levels in mandibular molars (gray) and maxillary molars (black), with values normalized with respect to *Gapdh* expression. Data (mean ±SEM) were analyzed with Student t-test; ***p<0.001; **p<0.01; *p<0.05.

To further validate our microarray experiments, a subset of 10 genes were selected for quantitative RT-PCR analysis. We focused our analysis on genes encoding known signaling molecules or their effectors: *Wnt11*, the FGF receptor gene *Fgfr4*, *Gli1* (an effector of the Hedgehog pathway), and *Dll1* (Delta-like 1) acting in the Notch pathway. We also chose the *Rorb* and *Cyp26c1* genes from the retinoic acid signaling pathway, and *Alx1* as a homeobox gene. We further decided to analyze one of the integrin genes (*Itga8*) identified as being differentially expressed, *Prkcq* (which was also found as differentially expressed between incisor and molar; see above), and *Adamtsl3*. Among the ten genes analyzed by qRT-PCR, eight were confirmed to be differentially expressed as detected by microarray analysis (Figure 4), whereas two (*Gli1* and *Wnt11*) were not found to be differentially expressed.

#### Gene network analysis

**Figure 5:**
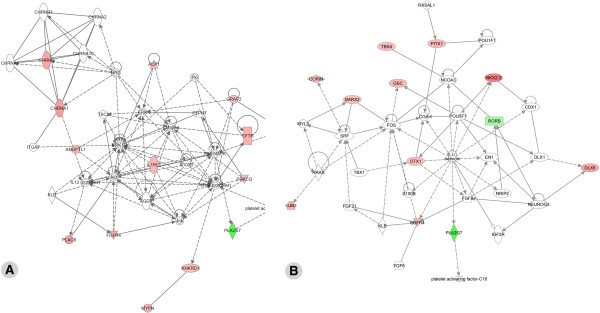
**Ingenuity pathway gene network analysis of maxillary (upper) vs. mandibular (lower) molar expressed genes. **The three most relevant networks identified by Ingenuity Pathway analysis are centered on ERK1/2 (**A**), and tretinoin (13-*cis*-retinoic acid, an active retinoid used in therapy) (**B**). All differentially expressed genes are shown in color, the intensity reflecting the degree of enrichment in mandibular (red) versus maxillary (green) molars. See Legend to Figure 3 for explanations on symbols and types of interactions.

**Relevant networks when considering all genes with a fold change higher than 2 **Among the 96 genes with a fold change higher than 2, 36 genes were mapped in two networks. The first network (score=27) was centered on ERK1/2 and included 13 genes identified as being differentially expressed between the two molar types (Figure 5A). *Chnrq, Chnra1, Acp1, Angtpl1, Plac8, Fgfr4, Il1r1, Grap2, Cftr, Prkcq, Ankrd1* and *Mypn* were more highly expressed in mandibular molars, whereas *Pla2g7* was enriched in maxillary molars. The second network (score=25) was centered on tretinoin (a retinoic acid derivative, used as a medication for skin diseases) and contained 12 differentially expressed genes (Figure 5B). *Rorb* and *Pla2g7* were the only two genes more expressed in maxillary molars, whereas *Pitx1*, *Tbx4*, *Gsc*, *Nkx2-3, Corin, Barx2, Otx1, Dlx6, Gjb2* and *Pgfr4* were more expressed in mandibular molars.

**Relevant networks when considering genes from selected pathways or families **From the 62 differentially expressed genes belonging to the pathways selected for analysis, 23 genes were mapped in only 2 different networks involved in embryonic or tissue development (Additional file 5). The first network contained 11 differentially expressed molecules and was centered on *Dlx1* (a gene known to be expressed in the presumptive molar region at E10.5 [17]. *Dlx1* and *Itga8* were the only two genes identified as being more highly expressed in maxillary molars, whereas *Nkx6-1, Phox2b, Gsx1, Rhox4b* were found to be enriched in mandibular molars. The second network contained 12 differentially expressed genes and was focused on *Gli3*. Only *Aldh1a1* was more expressed in maxillary molars, whereas *Barx2, Nkx3-2, Nkx2-1, Cer1, Lhx1, Gsc, Camk2b, Camk2a, Hmx2* and *Dll1* were preferentially expressed in mandibular molars.

## Conclusions

This study provides the first comprehensive analysis of differential gene expression between developing murine tooth types, leading to new insights into the regulatory mechanisms involved in the ontogenesis of mammalian teeth. Molecules belonging to pathways involved in various aspects of development (such as the Wnt, TGFβ/BMP, or FGF pathways) were discovered as potentially carrying information for differential tooth morphogenesis. Of interest is the involvement of the retinoic acid pathway [76], as retinoids have marked effects on molar and incisor morphogenesis [22,77]. Tooth morphology and its evolution in various mammalian species were proven to be related to dosage effect of signaling molecules, like for instance FGF3 being able to modify the cusps pattern [16,78]. Our microarray analysis highlighted molecules more or less strongly expressed in a given tooth type, reinforcing the model of dosage modulating mechanisms. Gene dosage abnormalities are likely to occur in human rare diseases presenting with a tooth family specific dental phenotype [37,38,79]. Some of the corresponding genes were not retrieved in our analysis of differential gene expression in lower incisors versus lower or upper molars, suggesting that other levels of regulation, post-transcriptionally via effectors of a given pathway or via fine tuning of kinase signaling (e.g. ref. [80]), will undoubtedly also participate in the molecular identity leading to specific tooth morphology. Future investigation of differential gene expressions between upper and lower incisors, two similar tooth types formed from neural crest cells of different origins, might also contribute to shed light on specific morphogenesis and its link to individual tooth shape.

## Methods

### Tissue collection

Pregnant C57BL6 female mice were euthanized at 14.5 days of gestation (E14.5), embryos were collected and tooth samples (lower incisors, mandibular and maxillary first molars) were microdissected. Tissue samples were frozen in liquid nitrogen and kept at -80°C until use. The CERBM-GIE/ICS/IGBMC complies with the French national and European laws and regulations relating to the transport, housing and use of animals in research.

### Microarray hybridization

Total RNA was extracted with the RNAeasy micro Kit (Qiagen) from pools of 4 tooth germs to obtain enough RNA for subsequent microarray hybridization. RNA quality was verified by analysis on a 2100 Bioanalyzer (Agilent). All samples displayed a RNA Integrity Number (RIN) greater than 9.8. Biotinylated single strand cDNA targets were prepared, starting from 300 ng of total RNA, using the Ambion WT Expression Kit (Cat #4411974) and the Affymetrix GeneChip WT Terminal Labeling Kit (Cat #900671), according to Affymetrix recommendations. Four lower incisors samples, 4 maxillary molars samples and 8 mandibular molars samples were hybridized on Affymetrix GeneChip Mouse Gene 1.0 ST arrays. Briefly, following fragmentation and end-labeling, 1.9 μg cDNA was hybridized for 16 h at 45°C on the arrays interrogating 28,853 genes represented by approximately 27 probes spread across the full length of the gene. The chips were washed and stained in the GeneChip Fluidics Station 450 (Affymetrix), and scanned with the GeneChip Scanner 3000 7G (Affymetrix). Finally, raw data (.CEL Intensity files) were extracted from the scanned images using the Affymetrix GeneChip Command Console (AGCC) version 3.1. One incisor sample was excluded from the analysis because a technical problem occured during hybridization washing.

### Microarray analysis

CEL files were further processed with the Partek software to obtain principal component analysis (PCA) and to select only genes with a signal value above 5 (20th percentile of all expression values) in at least one sample. The analysis was done only on three lower incisors samples as a technical problem during hybridization occurred for one of the 4 samples (high background). Genes were considered as differentially expressed if the false discovery rate from Benjamini and Hochberg test was under 0.1.

### Ingenuity pathways analysis

Biologically relevant networks were created using the Ingenuity Pathway Analysis software (http://www.Ingenuity.com). Based on the algorithmically generated connectivity between gene–gene, gene–protein, and protein–protein interactions, the program develops functional molecular networks that overlay genes in the dataset. This program calculated p-values for each network by comparing the number of genes that were mapped in a given network, relative to the total number of occurrences of those genes in all networks. The score for each network is given as the negative log of the p-value, which indicates the likelihood of finding a set of genes in the network by random chance. For instance, a score of 20 indicates that there is a 10^-20^ chance that the genes in focus would be in a network because of random chance. Networks taking in account direct and indirect interactions have been generated for genes with a fold change higher than 2, whereas networks involving only direct interactions have been created for genes that were selected as members of pathways or families of interest with a fold change higher than 1.2.

### Real-time quantitative RT-PCR

RT-PCR assays were performed in duplicate on three RNA samples for each tooth type, distinct from the ones used for microarray hybridization. RNA extractions were performed as previously described. Oligo-dT primed cDNAs were generated using the Superscript II kit (Invitrogen) according to the manufacturer’s protocol. Quantitative real-time PCR was achieved using SybrGreen and LightCycler 480 (Roche). The sequences of primers used for the various tested genes are given in Additional file 6. A probe set for detection of mouse *Gapdh* (a housekeeping gene) was used for normalisation. For each sample the ratio between signals for the gene of interest and *Gapdh* was calculated to normalize concentration values. To verify if genes were differentially expressed in different tooth types, the average of ratios calculated for lower incisors, mandibular molars and maxillary molars were then compared.

### Availability of supporting data

The data discussed in this publication have been deposited in NCBI's Gene Expression Omnibus (GEO) [81] and are accessible through GEO Series accession number GSE43144. (http://www.ncbi.nlm.nih.gov/geo/query/acc.cgi?acc=GSE43144).

## Competing interests

The authors declare that they have no competing interests.

## Authors’ contributions

All authors contributed to the overall experimental design. VLH and MP performed the tissue collection, RNA extraction, and real-time RT-PCR experiments. CTC did the microarray hybridization. VLH and DD performed all statistical analyses. VLH, PD and ABZ wrote the manuscript. All authors read, contributed to, and approved the final manuscript.

## Supplementary Material

Additional file 1 **Principal component analysis (PCA) of mandibular molar vs. lower incisor samples (A), and mandibular vs. maxillary molar samples (B). **Mandibular molar samples are represented in red, and incisor or maxillary molar samples in blue. The units are data-dependent and are generated by the software, which gives coordinates to each sample according to three axes that relate to the weight (inertia) of the decomposition into 3 principal components. For both analyses, samples segregate in two distinct groups, showing relevant transcriptional differences between the two tooth types. Click here for file

Additional file 2 **This table presents an overview of genes showing differential expression in developing mandibular incisors versus molars. **Only the genes exhibiting at least a two fold change in expression according to Affymetrix microarray analysis are listed. Genes with the highest expression in incisors (positive values) or molars (negative values) appear on top and bottom of the list, respectively. Click here for file

Additional file 3 **Ingenuity pathway gene network analysis of genes belonging to selected pathways and/or superfamily (homeobox genes), showing differential expression in incisor or molar tooth buds. **Four relevant networks were constructed by Ingenuity pathway analysis. The networks are displayed graphically as nodes (genes/gene products) and edges (biological relationships between the nodes). Differentially expressed genes are shown in two colors, the intensity of the colors reflecting the degree of enrichment in molar (red) versus incisor (green) tooth buds. Nodes are displayed using various shapes representing the functional class of the gene product (flat oval: transcription factor; tall oval: transmembrane receptor or interacting protein; losange: enzyme; triangle: kinase; rectangle: G protein-coupled receptor; circle: other). Interactions are depicted by arrows ("acts on", with dashed arrows indicating "indirect" interactions) or straight lines (binding only). Click here for file

Additional file 4 **Overview of genes showing differential expression in developing mandibular (lower) versus maxillary (upper) molars. **Only the genes exhibiting at least a two fold change in expression according to Affymetrix microarray analysis are listed. Genes with the highest expression in upper molars (positive values) or lower molars (negative values) appear on top and bottom of the list, respectively. Click here for file

Additional file 5 **Ingenuity pathway gene network analysis of genes belonging to selected pathways and/or superfamily (homeobox genes), showing differential expression in upper versus lower molars. **Two relevant networks are centered on *Dlx1* (network 1) and *Gli3 *(network 2). See Legend to Additional file 3 for key and explanations. Click here for file

Additional file 6 Sequences of primers used for real-time qRT-PCR assays. Click here for file
