# Supplementary figures and images for: Molars and incisors: show your microarray IDs
Source: BMC Res Notes. 2013 Mar 26;6:113. doi: 10.1186/1756-0500-6-113 (PMC3658942; doi:10.1186/1756-0500-6-113)

**Additional File 1**

**A**


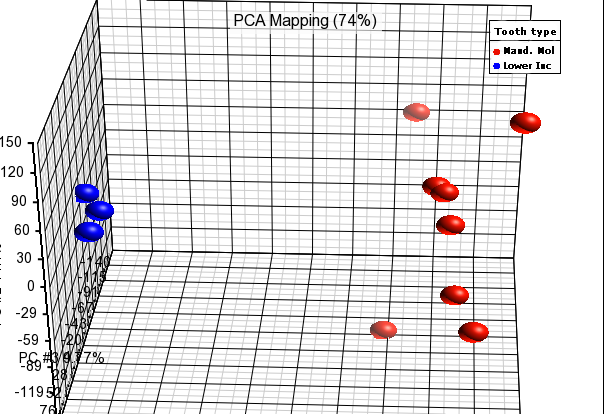


**B**


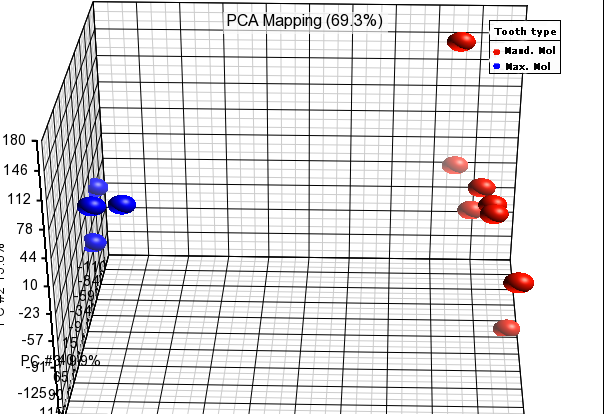

Supplement: Additional file 1 — Principal component analysis (PCA) of mandibular molar vs. lower incisor samples (A), and mandibular vs. maxillary molar samples (B). Mandibular molar samples are represented in red, and incisor or maxillary molar samples in blue. The units are data-dependent and are generated by the software, which gives coordinates to each sample according to three axes that relate to the weight (inertia) of the decomposition into 3 principal components. For both analyses, samples segregate in two distinct groups, showing relevant transcriptional differences between the two tooth types. [file 1756-0500-6-113-S1.docx]

**Additional File 3**

**Network 1**


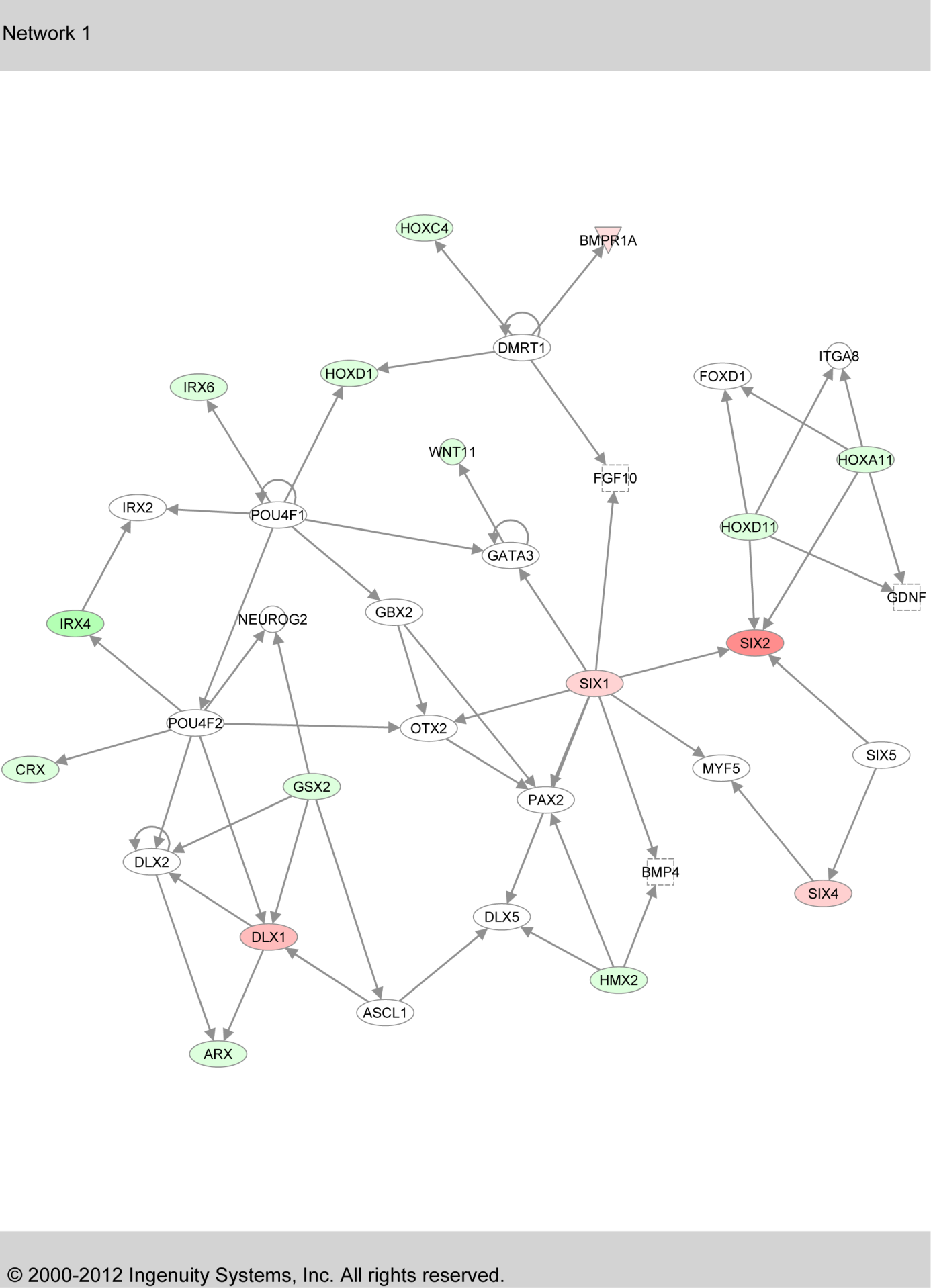


**Network 2**


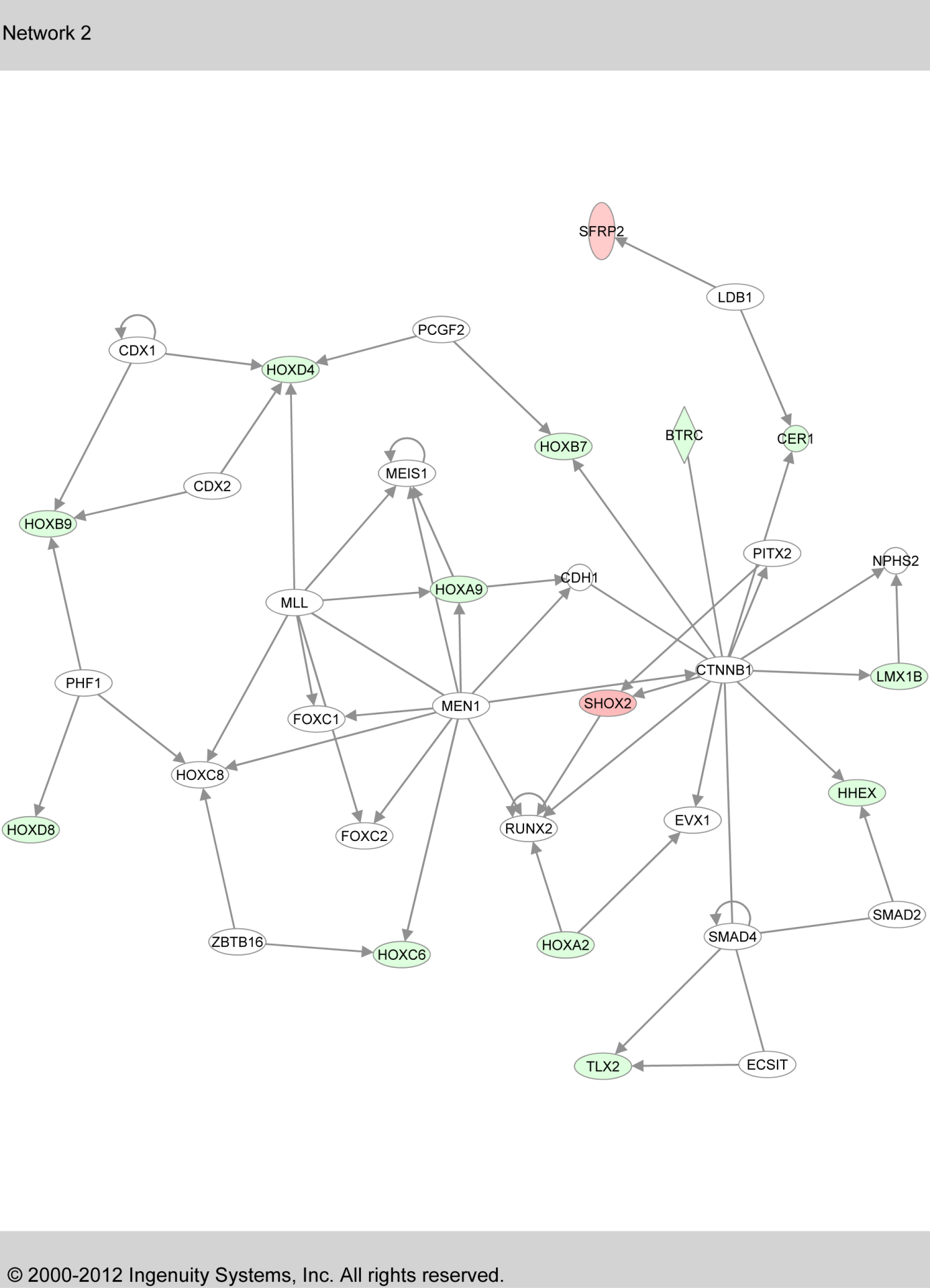


**Network 3**


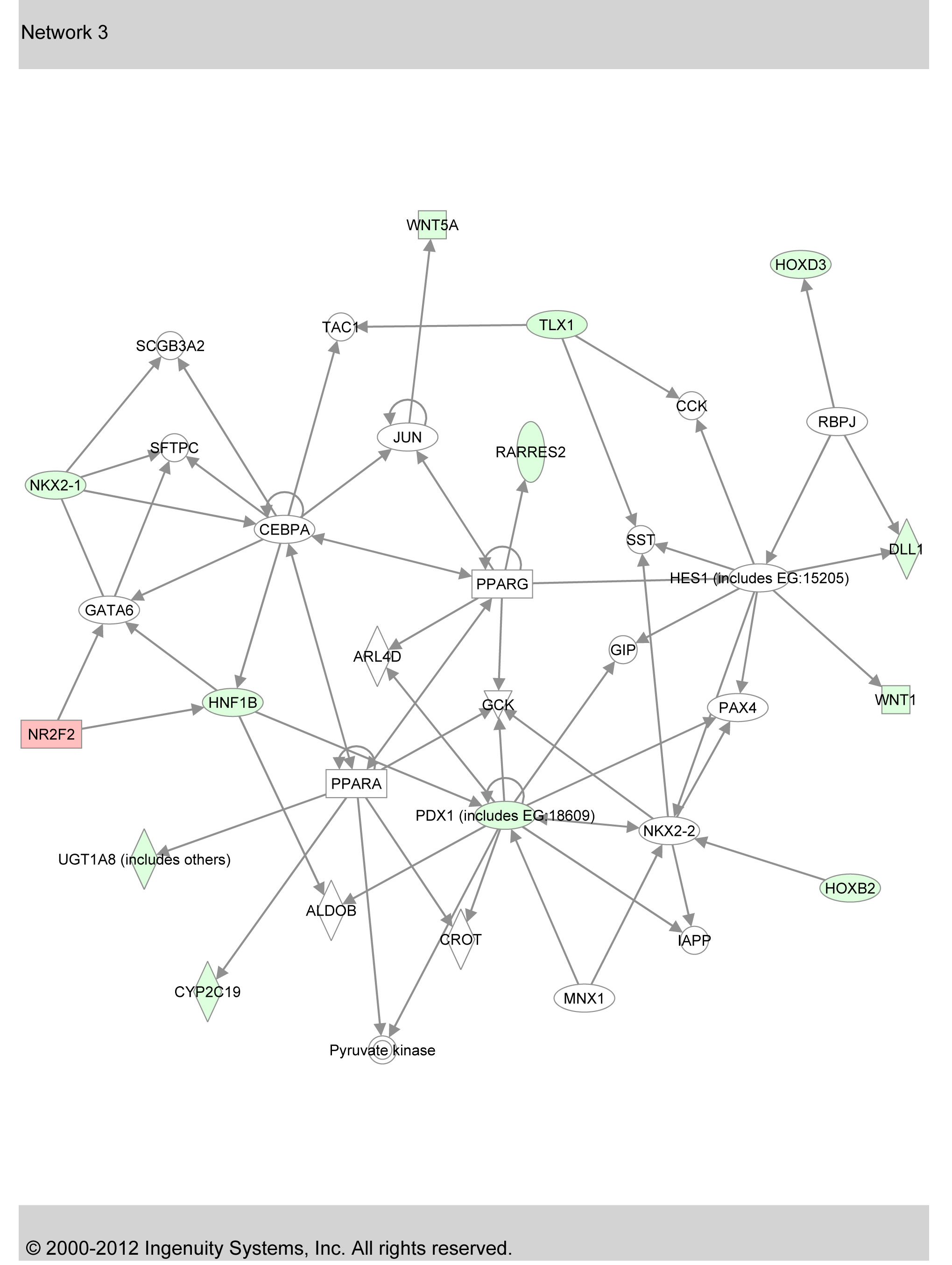


**Network 4**


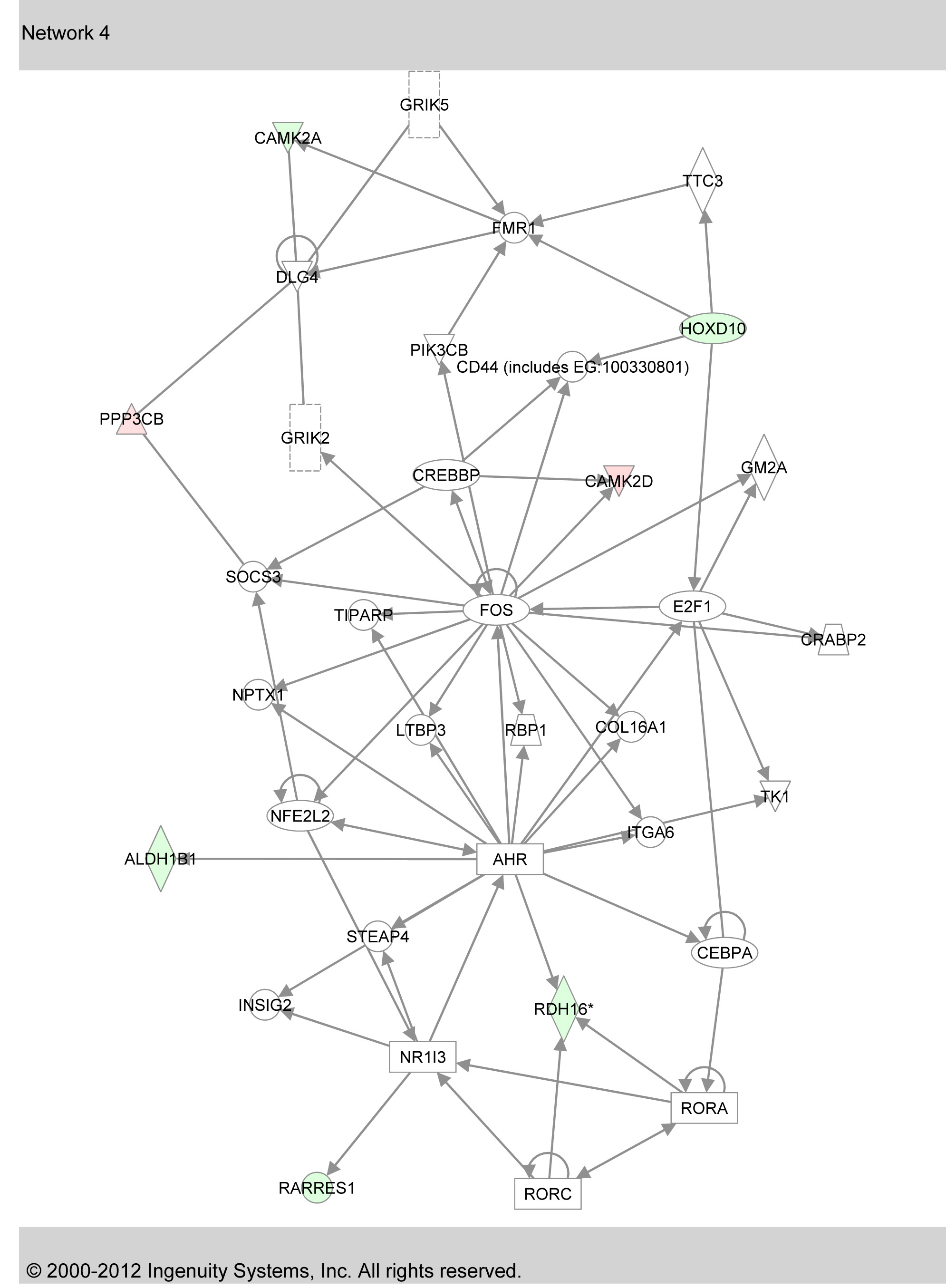

Supplement: Additional file 3 — Ingenuity pathway gene network analysis of genes belonging to selected pathways and/or superfamily (homeobox genes), showing differential expression in incisor or molar tooth buds. Four relevant networks were constructed by Ingenuity pathway analysis. The networks are displayed graphically as nodes (genes/gene products) and edges (biological relationships between the nodes). Differentially expressed genes are shown in two colors, the intensity of the colors reflecting the degree of enrichment in molar (red) versus incisor (green) tooth buds. Nodes are displayed using various shapes representing the functional class of the gene product (flat oval: transcription factor; tall oval: transmembrane receptor or interacting protein; losange: enzyme; triangle: kinase; rectangle: G protein-coupled receptor; circle: other). Interactions are depicted by arrows ("acts on", with dashed arrows indicating "indirect" interactions) or straight lines (binding only). [file 1756-0500-6-113-S3.docx]

**Network 1**

**Additional File 5**


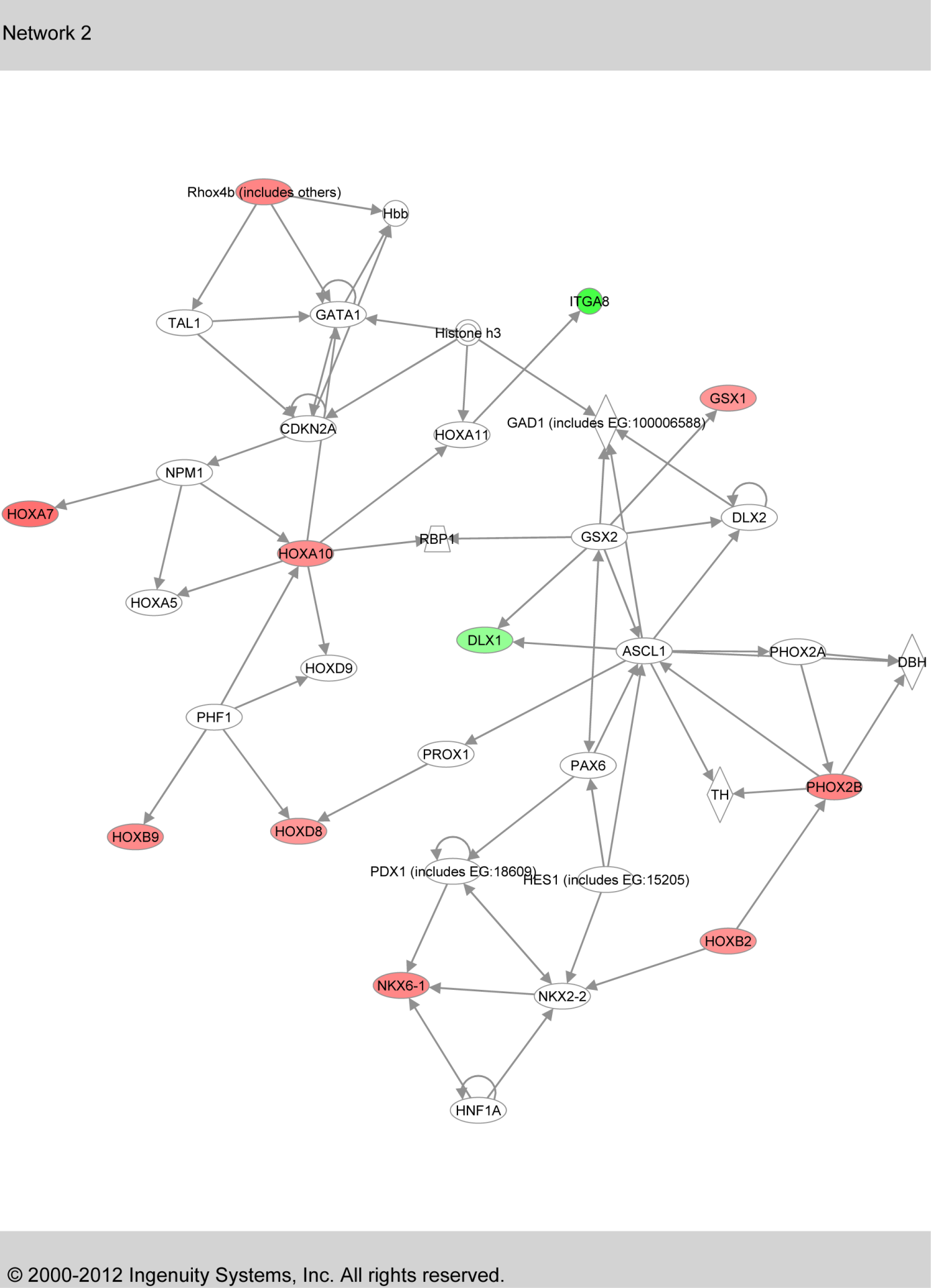


**Network 2**


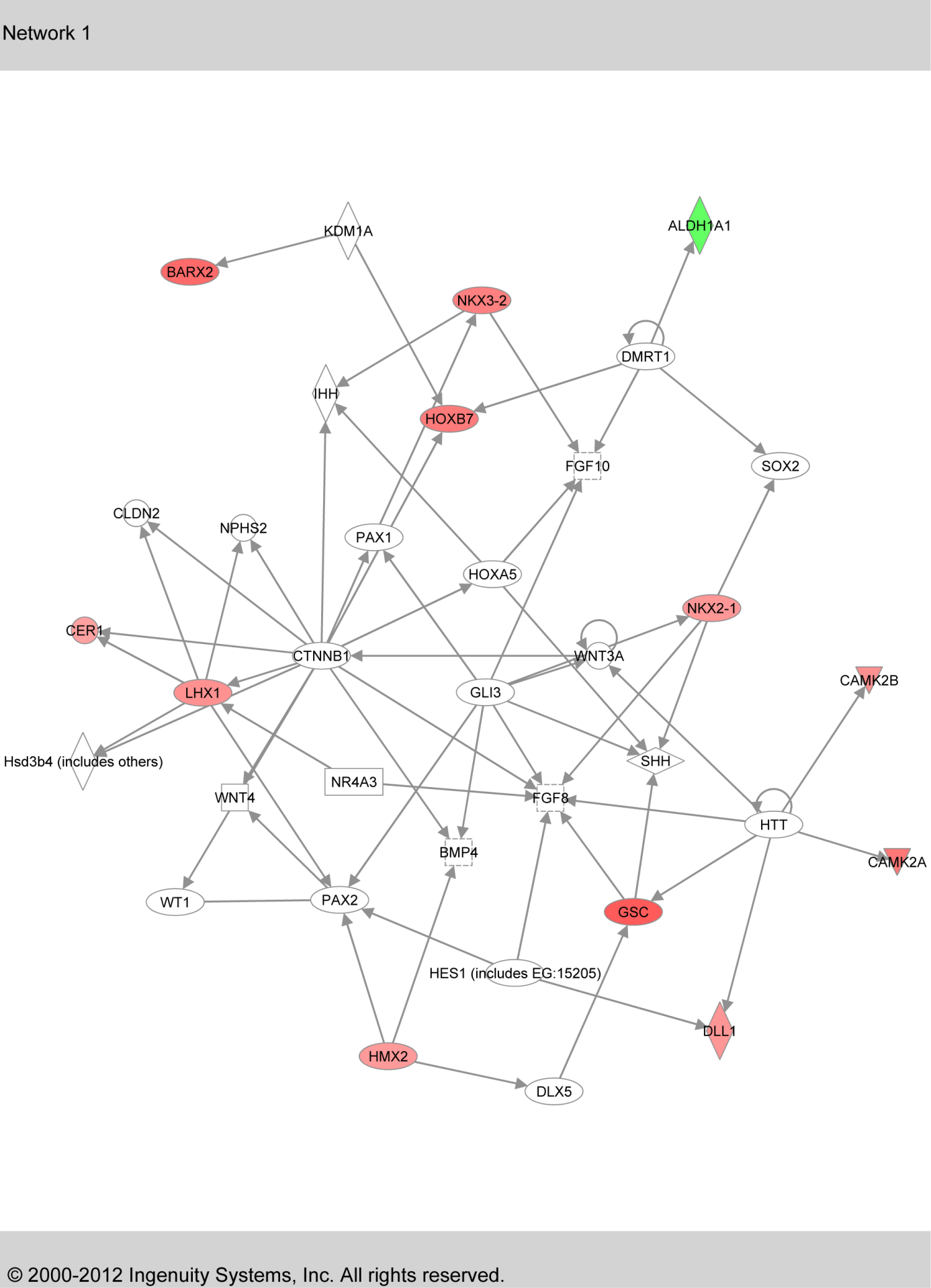

Supplement: Additional file 5 — Ingenuity pathway gene network analysis of genes belonging to selected pathways and/or superfamily (homeobox genes), showing differential expression in upper versus lower molars. Two relevant networks are centered on Dlx1 (network 1) and Gli3 (network 2). See Legend to Additional file 3 for key and explanations. [file 1756-0500-6-113-S5.docx]
