# Supplementary material for: Molars and incisors: show your microarray IDs
Source: BMC Res Notes. 2013 Mar 26;6:113. doi: 10.1186/1756-0500-6-113 (PMC3658942; doi:10.1186/1756-0500-6-113)
Supplement: Additional file 2 — This table presents an overview of genes showing differential expression in developing mandibular incisors versus molars. Only the genes exhibiting at least a two fold change in expression according to Affymetrix microarray analysis are listed. Genes with the highest expression in incisors (positive values) or molars (negative values) appear on top and bottom of the list, respectively. [file 1756-0500-6-113-S2.docx]

**Additional File 2**

| **Gene Symbol** | **Gene Description** | **GO Cellular Component Term** | **GO Molecular Function Term** | **p-value** | **Fold change** |
| --- | --- | --- | --- | --- | --- |
| ***Barx1*** | BarH-like homeobox 1 | nucleus | transcription factor activity | 3,47E-09 | 8,60 |
| ***C1qtnf3*** | C1q and tumor necrosis factor related protein 3 | extracellular region | not determined | 4,37E-08 | 7,30 |
| ***Adcy8*** | adenylate cyclase 8 | membrane fraction | adenylate cyclase activity | 2,76E-08 | 5,75 |
| ***Cntn6*** | contactin 6 | plasma membrane | protein binding | 2,94E-07 | 4,91 |
| ***Six2*** | sine oculis-related homeobox 2 homolog (Drosophila) | nucleus | transcription factor activity | 4,84E-08 | 4,82 |
| ***Tcfap2b*** | transcription factor AP-2 beta | nucleus | transcription factor activity | 1,88E-05 | 4,31 |
| ***Odz1*** | odd Oz/ten-m homolog 1 (Drosophila) | integral to plasma membrane | --- | 5,02E-09 | 4,24 |
| ***Vstm2a*** | V-set and transmembrane domain containing 2A | not determined | not determined | 7,28E-08 | 4,09 |
| ***Nptx1*** | neuronal pentraxin 1 | cytoplasmic vesicle | metal ion binding | 1,21E-05 | 4,06 |
| ***Has2*** | hyaluronan synthase 2 | membrane | transferase activity, transferring glycosyl groups | 1,66E-09 | 3,94 |
| ***Lhx6*** | LIM homeobox protein 6 | nucleus | DNA binding | 8,95E-08 | 3,69 |
| ***Cyp1b1*** | cytochrome P450, family 1, subfamily b, polypeptide 1 | endoplasmic reticulum | iron ion binding | 9,56E-08 | 3,60 |
| ***Nr2f1*** | nuclear receptor subfamily 2, group F, member 1 | nucleus | transcription factor activity | 2,24E-06 | 3,59 |
| ***Sfrp1*** | secreted frizzled-related protein 1 | extracellular region | protein binding | 7,94E-06 | 3,56 |
| ***Rgs5*** | regulator of G-protein signaling 5 | --- | signal transducer activity | 1,15E-08 | 3,45 |
| ***Itgbl1*** | integrin, beta-like 1 | not determined | receptor activity // | 8,44E-07 | 3,42 |
| ***Slc25a21*** | solute carrier family 25 (mitochondrial oxodicarboxylate carrier), member 21 | mitochondrion | binding | 8,03E-07 | 3,32 |
| ***Ncam2*** | neural cell adhesion molecule 2 | plasma membrane | protein binding | 1,72E-07 | 3,31 |
| ***Smoc2*** | SPARC related modular calcium binding 2 | extracellular region | glycosaminoglycan binding | 3,28E-08 | 3,29 |
| ***Nxph1*** | neurexophilin 1 | extracellular region | receptor binding | 2,78E-09 | 3,18 |
| ***Grp*** | gastrin releasing peptide | extracellular region | neuropeptide hormone activity | 2,71E-08 | 3,11 |
| ***Gria1*** | glutamate receptor, ionotropic, AMPA1 (alpha 1) | membrane fraction | ionotropic glutamate receptor activity | 9,28E-08 | 3,01 |
| ***Ppfia2*** | protein tyrosine phosphatase, receptor type, f polypeptide (PTPRF), interacting protein (liprin), alpha 2 | cytoplasm | protein binding | 8,06E-09 | 2,96 |
| ***Tmem30b*** | transmembrane protein 30B | not determined | not determined | 3,07E-08 | 2,90 |
| ***Shox2*** | short stature homeobox 2 | nucleus | transcription factor activity | 1,08E-08 | 2,85 |
| ***Dgkb*** | diacylglycerol kinase, beta | membrane fraction | diacylglycerol kinase activity | 3,70E-07 | 2,82 |
| ***Dlx1*** | distal-less homeobox 1 | nucleus | chromatin binding | 4,49E-11 | 2,81 |
| ***Slc5a7*** | solute carrier family 5 (choline transporter), member 7 | plasma membrane | choline:sodium symporter activity | 2,51E-07 | 2,81 |
| ***Kera*** | keratocan | extracellular region | protein binding | 9,92E-06 | 2,80 |
| ***Vsnl1*** | visinin-like 1 | --- | calcium ion binding | 1,02E-05 | 2,76 |
| ***Gabra1*** | gamma-aminobutyric acid (GABA) A receptor, subunit alpha 1 | plasma membrane | GABA-A receptor activity | 8,46E-09 | 2,75 |
| ***2900055J20Rik*** | RIKEN cDNA 2900055J20 gene | not determined | not determined | 5,19E-07 | 2,74 |
| ***Gabrb2*** | gamma-aminobutyric acid (GABA) A receptor, subunit beta 2 | membrane fraction | ion channel activity | 2,37E-07 | 2,72 |
| ***Sox2*** | SRY-box containing gene 2 | nucleus | DNA binding | 2,80E-06 | 2,69 |
| ***Nr2f2*** | nuclear receptor subfamily 2, group F, member 2 | nucleus | transcription factor activity | 9,19E-09 | 2,69 |
| ***Entpd1*** | ectonucleoside triphosphate diphosphohydrolase 1 | basal lamina | protein binding | 1,47E-06 | 2,66 |
| ***Nup62cl*** | nucleoporin 62 C-terminal like | not determined | not determined | 4,14E-09 | 2,64 |
| ***Clstn2*** | calsyntenin 2 | endoplasmic reticulum | calcium ion binding // protein binding | 2,95E-10 | 2,64 |
| ***Galnt13*** | UDP-N-acetyl-alpha-D-galactosamine:polypeptide N-acetylgalactosaminyltransferase 13 | Golgi apparatus | sugar binding | 6,92E-06 | 2,57 |
| ***Cysltr1*** | cysteinyl leukotriene receptor 1 | plasma membrane | receptor activity | 4,02E-08 | 2,56 |
| ***Bmper*** | BMP-binding endothelial regulator | extracellular region | protein binding | 1,06E-06 | 2,56 |
| ***Dusp6*** | dual specificity phosphatase 6 | cytoplasm | protein tyrosine phosphatase activity | 7,62E-06 | 2,52 |
| ***Cxcl5*** | chemokine (C-X-C motif) ligand 5 | extracellular region | chemokine activity | 4,59E-07 | 2,51 |
| ***Nell1*** | NEL-like 1 (chicken) | extracellular region | structural molecule activity | 2,27E-06 | 2,51 |
| ***AI427809*** | expressed sequence AI427809 | not determined | not determined | 1,44E-08 | 2,50 |
| ***Pax9*** | paired box gene 9 | nucleus | transcription factor activity | 2,66E-07 | 2,49 |
| ***Gabra2*** | gamma-aminobutyric acid (GABA) A receptor, subunit alpha 2 | plasma membrane | GABA-A receptor activity | 2,41E-07 | 2,46 |
| ***Vps33b*** | vacuolar protein sorting 33B (yeast) | not determined | syntaxin-2 binding | 1,32E-07 | 2,41 |
| ***Leprel1*** | leprecan-like 1 | endoplasmic reticulum | oxidoreductase activity | 9,24E-08 | 2,38 |
| ***Calml4*** | calmodulin-like 4 | --- | calcium ion binding | 1,94E-06 | 2,35 |
| ***Msr1*** | macrophage scavenger receptor 1 | cytosol | scavenger receptor activity | 3,83E-08 | 2,33 |
| ***Cdh12*** | cadherin 12 | not determined | calcium ion binding | 1,69E-05 | 2,32 |
| ***Ptchd1*** | patched domain containing 1 | not determined | hedgehog receptor activity | 1,22E-08 | 2,27 |
| ***Fgf12*** | fibroblast growth factor 12 | nucleus | protein binding | 6,11E-08 | 2,24 |
| ***Slitrk5*** | SLIT and NTRK-like family, member 5 | membrane | protein binding | 4,65E-08 | 2,22 |
| ***Rgs5*** | regulator of G-protein signaling 5 | --- | GTPase activator activity | 3,32E-06 | 2,22 |
| ***Cldn10a*** | claudin 10A | plasma membrane | structural molecule activity | 1,11E-05 | 2,21 |
| ***Ucma*** | upper zone of growth plate and cartilage matrix associated | extracellular region | --- | 4,13E-06 | 2,20 |
| ***Dach1*** | dachshund 1 (Drosophila) | nucleus | DNA binding | 2,48E-07 | 2,20 |
| ***Gria2*** | glutamate receptor, ionotropic, AMPA2 (alpha 2) | membrane fraction | ionotropic glutamate receptor activity | 1,46E-05 | 2,18 |
| ***Urb2*** | URB2 ribosome biogenesis 2 homolog (S. cerevisiae) | not determined | not determined | 5,89E-06 | 2,18 |
| ***Pid1*** | phosphotyrosine interaction domain containing 1 | not determined | not determined | 5,00E-07 | 2,16 |
| ***Sfrp2*** | secreted frizzled-related protein 2 | extracellular region | Wnt-protein binding | 4,40E-11 | 2,16 |
| ***Cited1*** | Cbp/p300-interacting transactivator with Glu/Asp-rich carboxy-terminal domain 1 | nucleus | protein binding | 2,69E-07 | 2,16 |
| ***Ccdc53*** | coiled-coil domain containing 53 | not determined | not determined | 5,38E-08 | 2,15 |
| ***Lpl*** | lipoprotein lipase | extracellular space | lipoprotein lipase activity | 8,09E-08 | 2,13 |
| ***Nlgn1*** | neuroligin 1 | plasma membrane | protein binding | 1,04E-07 | 2,11 |
| ***Scd3*** | stearoyl-coenzyme A desaturase 3 | integral to membrane | oxidoreductase activity | 5,58E-08 | 2,11 |
| ***Myo9a*** | myosin IXa | intracellular | motor activity | 1,64E-07 | 2,11 |
| ***Nmbr*** | neuromedin B receptor | plasma membrane | receptor activity | 2,01E-06 | 2,09 |
| ***Prr15*** | proline rich 15 | not determined | not determined | 2,22E-07 | 2,09 |
| ***Cdc40*** | cell division cycle 40 homolog (yeast) | not determined | not determined | 5,22E-08 | 2,09 |
| ***Lrrtm1*** | leucine rich repeat transmembrane neuronal 1 | endoplasmic reticulum | protein binding | 1,12E-06 | 2,09 |
| ***Bard1*** | BRCA1 associated RING domain 1 | intracellular | protein binding | 1,13E-05 | 2,07 |
| ***Hdac9*** | histone deacetylase 9 | histone deacetylase complex | histone deacetylase activity | 7,24E-06 | 2,05 |
| ***Man1a*** | mannosidase 1, alpha | Golgi membrane | calcium ion binding | 3,15E-06 | 2,03 |
| ***Npas3*** | neuronal PAS domain protein 3 | nucleus | signal transducer activity | 8,60E-06 | 2,02 |
| ***Ror1*** | receptor tyrosine kinase-like orphan receptor 1 | integral to plasma membrane | protein kinase activity | 9,37E-09 | 2,01 |
| ***Mgat4a*** | mannoside acetylglucosaminyltransferase 4, isoenzyme A | extracellular region | transferase activity | 2,01E-06 | 2,01 |
| ***Sdcbp2*** | syndecan binding protein (syntenin) 2 | not determined | protein binding | 5,30E-09 | -2,00 |
| ***Fam69c*** | family with sequence similarity 69, member C | not determined | not determined | 3,03E-06 | -2,00 |
| ***Hmcn1*** | hemicentin 1 | extracellular region | not determined | 2,59E-06 | -2,00 |
| ***Mrgprd*** | MAS-related GPR, member D | plasma membrane | receptor activity | 1,36E-09 | -2,00 |
| ***Foxa3*** | forkhead box A3 | nucleus | DNA binding | 3,73E-09 | -2,00 |
| ***Mir200c*** | microRNA 200c | --- | --- | 4,70E-06 | -2,00 |
| ***Gm16432*** | predicted gene 16432 | not determined | not determined | 4,61E-08 | -2,00 |
| ***Gm16498*** | predicted gene 16498 | not determined | not determined | 4,12E-06 | -2,01 |
| ***Cma2*** | chymase 2, mast cell | not determined | peptidase activity | 7,24E-10 | -2,01 |
| ***A430089I19Rik*** | RIKEN cDNA A430089I19 gene | not determined | not determined | 2,25E-08 | -2,02 |
| ***A430089I19Rik*** | RIKEN cDNA A430089I19 gene | not determined | not determined | 2,25E-08 | -2,02 |
| ***Epyc*** | epiphycan | extracellular region | protein binding | 3,88E-07 | -2,03 |
| ***Ccdc81*** | coiled-coil domain containing 81 | not determined | not determined | 7,88E-09 | -2,03 |
| ***Rgs20*** | regulator of G-protein signaling 20 | nucleus | GTPase activator activity | 3,41E-10 | -2,04 |
| ***4921508D12Rik*** | RIKEN cDNA 4921508D12 gene | not determined | not determined | 2,81E-10 | -2,04 |
| ***Fam163a*** | family with sequence similarity 163, member A | not determined | not determined | 6,72E-07 | -2,04 |
| ***Gm10385*** | predicted gene 10385 | not determined | not determined | 7,22E-09 | -2,04 |
| ***Gm10210*** | predicted gene 10210 | --- | --- | 8,92E-06 | -2,04 |
| ***Cyp17a1*** | cytochrome P450, family 17, subfamily a, polypeptide 1 | mitochondrion | steroid 17-alpha-monooxygenase activity | 8,36E-10 | -2,05 |
| ***Gm10304*** | predicted gene 10304 | not determined | not determined | 1,72E-06 | -2,05 |
| ***Olfr46*** | olfactory receptor 46 | integral to membrane | receptor activity | 7,09E-09 | -2,05 |
| ***Gm9276*** | eukaryotic translation elongation factor 1 gamma pseudogene | --- | --- | 7,67E-07 | -2,05 |
| ***Tac2*** | tachykinin 2 | extracellular region | --- | 1,02E-06 | -2,05 |
| ***Fam155a*** | family with sequence similarity 155, member A | not determined | not determined | 1,59E-10 | -2,06 |
| ***Vmn2r51*** | vomeronasal 2, receptor 51 | not determined | not determined | 5,04E-06 | -2,06 |
| ***Il16*** | interleukin 16 | extracellular region | protein binding | 3,84E-06 | -2,06 |
| ***Olfr810*** | olfactory receptor 810 | integral to membrane | receptor activity | 4,38E-09 | -2,06 |
| ***Gm7849*** | predicted gene 7849 | not determined | not determined | 2,55E-06 | -2,06 |
| ***5930412G12Rik*** | RIKEN cDNA 5930412G12 gene | not determined | not determined | 2,18E-09 | -2,06 |
| ***D830046C22Rik*** | RIKEN cDNA D830046C22 gene | not determined | not determined | 1,36E-09 | -2,07 |
| ***Cma1*** | chymase 1, mast cell | extracellular region | serine-type endopeptidase activity | 1,56E-07 | -2,07 |
| ***Olfr698*** | olfactory receptor 698 | integral to membrane | olfactory receptor activity | 1,24E-11 | -2,07 |
| ***Smok2b*** | sperm motility kinase 2B | not determined | not determined | 1,41E-08 | -2,07 |
| ***Wscd2*** | WSC domain containing 2 | not determined | not determined | 4,62E-10 | -2,07 |
| ***4930467E23Rik*** | RIKEN cDNA 4930467E23 gene | not determined | not determined | 8,03E-06 | -2,07 |
| ***Gm2736*** | predicted gene 2736 | --- | --- | 8,56E-08 | -2,08 |
| ***F830212C03Rik*** | RIKEN cDNA F830212C03 gene | not determined | not determined | 5,08E-07 | -2,08 |
| ***A430089I19Rik*** | RIKEN cDNA A430089I19 gene | not determined | not determined | 7,87E-10 | -2,08 |
| ***A430089I19Rik*** | RIKEN cDNA A430089I19 gene | not determined | not determined | 7,87E-10 | -2,08 |
| ***Reln*** | reelin | extracellular region | protein binding | 5,84E-06 | -2,08 |
| ***Pou2f3*** | POU domain, class 2, transcription factor 3 | nucleus | transcription regulator activity | 1,45E-05 | -2,09 |
| ***Wdr64*** | WD repeat domain 64 | not determined | not determined | 1,51E-08 | -2,10 |
| ***A430089I19Rik*** | RIKEN cDNA A430089I19 gene | not determined | not determined | 4,57E-09 | -2,10 |
| ***1700026D08Rik*** | RIKEN cDNA 1700026D08 gene | not determined | not determined | 1,60E-08 | -2,11 |
| ***Gm7735*** | predicted gene 7735 | not determined | not determined | 1,45E-08 | -2,11 |
| ***4930467E23Rik*** | RIKEN cDNA 4930467E23 gene | not determined | not determined | 3,28E-07 | -2,11 |
| ***Lrrn3*** | leucine rich repeat protein 3, neuronal | membrane | protein binding | 7,55E-08 | -2,12 |
| ***C1qtnf2*** | C1q and tumor necrosis factor related protein 2 | extracellular region | receptor binding | 5,88E-08 | -2,12 |
| ***Rbpjl*** | recombination signal binding protein for immunoglobulin kappa J region-like | nucleus | transcription factor activity | 1,37E-07 | -2,12 |
| ***A430089I19Rik*** | RIKEN cDNA A430089I19 gene | not determined | not determined | 5,43E-10 | -2,13 |
| ***Clec2h*** | C-type lectin domain family 2, member h | plasma membrane | transmembrane receptor activity | 1,89E-07 | -2,13 |
| ***Gm7174*** | predicted gene 7174 | not determined | not determined | 4,87E-07 | -2,14 |
| ***Epgn*** | epithelial mitogen | membrane | epidermal growth factor receptor binding | 3,30E-08 | -2,15 |
| ***4930467E23Rik*** | RIKEN cDNA 4930467E23 gene | not determined | not determined | 1,75E-06 | -2,15 |
| ***Ins1*** | insulin I | extracellular region | insulin receptor binding | 3,70E-10 | -2,15 |
| ***5330417C22Rik*** | RIKEN cDNA 5330417C22 gene | not determined | protein binding | 2,07E-06 | -2,16 |
| ***Olfr1200*** | olfactory receptor 1200 | integral to membrane | receptor activity | 2,29E-07 | -2,16 |
| ***Aqp1*** | aquaporin 1 | integral to membrane of membrane fraction | water transmembrane transporter activity | 9,51E-08 | -2,16 |
| ***E030019B06Rik*** | RIKEN cDNA E030019B06 gene | not determined | not determined | 3,49E-10 | -2,16 |
| ***A430089I19Rik*** | RIKEN cDNA A430089I19 gene | not determined | not determined | 1,84E-09 | -2,17 |
| ***A430089I19Rik*** | RIKEN cDNA A430089I19 gene | not determined | not determined | 1,84E-09 | -2,17 |
| ***A430089I19Rik*** | RIKEN cDNA A430089I19 gene | not determined | not determined | 1,84E-09 | -2,17 |
| ***Olfr878*** | olfactory receptor 878 | integral to membrane | receptor activity | 9,41E-10 | -2,17 |
| ***Ly6g6c*** | lymphocyte antigen 6 complex, locus G6C | not determined | not determined | 3,95E-06 | -2,18 |
| ***Gzme*** | granzyme E | --- | serine-type endopeptidase activity | 4,39E-07 | -2,19 |
| ***Masp1*** | mannan-binding lectin serine peptidase 1 | extracellular region | serine-type endopeptidase activity | 3,74E-07 | -2,19 |
| ***4930467E23Rik*** | RIKEN cDNA 4930467E23 gene | not determined | not determined | 1,45E-06 | -2,19 |
| ***Dnahc3*** | dynein, axonemal, heavy chain 3 | not determined | not determined | 1,95E-07 | -2,19 |
| ***Tgs1*** | trimethylguanosine synthase homolog (S. cerevisiae) | nucleus | methyltransferase activity | 1,03E-06 | -2,19 |
| ***Scara5*** | scavenger receptor class A, member 5 (putative) | plasma membrane | scavenger receptor activity | 3,96E-07 | -2,21 |
| ***1700029M20Rik*** | RIKEN cDNA 1700029M20 gene | not determined | not determined | 2,04E-07 | -2,22 |
| ***Kcnh7*** | potassium voltage-gated channel, subfamily H (eag-related), member 7 | not determined | inward rectifier potassium channel activity | 3,28E-08 | -2,23 |
| ***Dio2*** | deiodinase, iodothyronine, type II | membrane | thyroxine 5'-deiodinase activity | 2,90E-08 | -2,23 |
| ***Kcna1*** | potassium voltage-gated channel, shaker-related subfamily, member 1 | voltage-gated potassium channel complex | voltage-gated ion channel activity | 6,84E-07 | -2,24 |
| ***Nrn1*** | neuritin 1 | plasma membrane | --- | 1,04E-05 | -2,25 |
| ***9630013D21Rik*** | RIKEN cDNA 9630013D21 gene | not determined | not determined | 9,60E-10 | -2,26 |
| ***Cyp2c54*** | cytochrome P450, family 2, subfamily c, polypeptide 54 | endoplasmic reticulum | iron ion binding | 4,98E-08 | -2,26 |
| ***Serpinb9e*** | serine (or cysteine) peptidase inhibitor, clade B, member 9e | not determined | not determined | 1,87E-08 | -2,27 |
| ***Amtn*** | amelotin | extracellular region | protein binding | 1,65E-08 | -2,28 |
| ***6330403A02Rik*** | RIKEN cDNA 6330403A02 gene | not determined | not determined | 9,87E-09 | -2,28 |
| ***Bpil2*** | bactericidal/permeability-increasing protein-like 2 | not determined | lipid binding | 1,48E-07 | -2,29 |
| ***Mug1*** | murinoglobulin 1 | extracellular region | serine-type endopeptidase inhibitor activity | 1,91E-10 | -2,29 |
| ***Prtg*** | protogenin homolog (Gallus gallus) | membrane | --- | 9,93E-07 | -2,30 |
| ***Lman1l*** | lectin, mannose-binding 1 like | not determined | sugar binding | 1,23E-09 | -2,32 |
| ***Gm10664*** | predicted gene 10664 | not determined | not determined | 6,70E-06 | -2,33 |
| ***Lrrc4*** | leucine rich repeat containing 4 | plasma membrane | protein binding | 2,16E-06 | -2,34 |
| ***2810055G20Rik*** | RIKEN cDNA 2810055G20 gene | not determined | not determined | 3,08E-09 | -2,36 |
| ***Pappa*** | pregnancy-associated plasma protein A | extracellular region | peptidase activity | 3,32E-07 | -2,37 |
| ***Slitrk6*** | SLIT and NTRK-like family, member 6 | membrane | protein binding | 2,60E-07 | -2,37 |
| ***Qrfpr*** | pyroglutamylated RFamide peptide receptor | plasma membrane | receptor activity | 1,38E-06 | -2,37 |
| ***Wnt5a*** | wingless-related MMTV integration site 5A | extracellular region | receptor binding | 1,38E-05 | -2,38 |
| ***Tceal6*** | transcription elongation factor A (SII)-like 6 | not determined | translation elongation factor activity | 3,51E-08 | -2,40 |
| ***Glis3*** | GLIS family zinc finger 3 | intracellular | DNA binding | 7,34E-07 | -2,40 |
| ***Nlrp5*** | NLR family, pyrin domain containing 5 | nucleus | protein binding | 6,94E-10 | -2,40 |
| ***4930467E23Rik*** | RIKEN cDNA 4930467E23 gene | not determined | not determined | 6,58E-09 | -2,44 |
| ***Glis1*** | GLIS family zinc finger 1 | intracellular | DNA binding | 6,78E-06 | -2,47 |
| ***Cacna1d*** | calcium channel, voltage-dependent, L type, alpha 1D subunit | plasma membrane | voltage-gated ion channel activity | 1,25E-09 | -2,47 |
| ***Ppargc1a*** | peroxisome proliferative activated receptor, gamma, coactivator 1 alpha | nucleus | nucleic acid binding | 2,22E-06 | -2,48 |
| ***Ptprr*** | protein tyrosine phosphatase, receptor type, R | cytoplasm | protein tyrosine phosphatase activity | 1,45E-06 | -2,48 |
| ***Ctnna2*** | catenin (cadherin associated protein), alpha 2 | cytoplasm | protein binding | 3,76E-08 | -2,48 |
| ***Otx1*** | orthodenticle homolog 1 (Drosophila) | nucleus | transcription factor activity | 5,56E-07 | -2,48 |
| ***Olfr1126*** | olfactory receptor 1126 | integral to membrane | receptor activity | 2,46E-12 | -2,49 |
| ***AI593442*** | expressed sequence AI593442 | not determined | not determined | 2,49E-06 | -2,50 |
| ***Klhl14*** | kelch-like 14 (Drosophila) | not determined | not determined | 4,34E-09 | -2,51 |
| ***Ace2*** | angiotensin I converting enzyme (peptidyl-dipeptidase A) 2 | extracellular region | carboxypeptidase activity | 3,02E-09 | -2,52 |
| ***Ramp3*** | receptor (calcitonin) activity modifying protein 3 | membrane | protein transporter activity | 1,28E-06 | -2,53 |
| ***Pla2g2f*** | phospholipase A2, group IIF | extracellular region | calcium ion binding | 2,78E-10 | -2,54 |
| ***Hormad1*** | HORMA domain containing 1 | not determined | not determined | 1,15E-09 | -2,54 |
| ***Adamts16*** | a disintegrin-like and metallopeptidase (reprolysin type) with thrombospondin type 1 motif, 16 | not determined | metalloendopeptidase activity | 2,12E-07 | -2,54 |
| ***Hand1*** | heart and neural crest derivatives expressed transcript 1 | nucleus | transcription factor activity | 4,01E-06 | -2,55 |
| ***Olfr166*** | olfactory receptor 166 | integral to membrane | receptor activity | 1,42E-08 | -2,57 |
| ***Cyp4f39*** | cytochrome P450, family 4, subfamily f, polypeptide 39 | not determined | monooxygenase activity | 1,40E-06 | -2,58 |
| ***Sprr1b*** | small proline-rich protein 1B | cornified envelope | structural constituent of cytoskeleton | 1,69E-09 | -2,60 |
| ***Olfr1043*** | olfactory receptor 1043 | integral to membrane | receptor activity | 7,07E-12 | -2,64 |
| ***Syt17*** | synaptotagmin XVII | trans-Golgi network | transporter activity | 9,08E-11 | -2,67 |
| ***Unc5c*** | unc-5 homolog C (C. elegans) | plasma membrane | netrin receptor activity | 2,98E-07 | -2,68 |
| ***Prkcq*** | protein kinase C, theta | immunological synapse | protein kinase activity | 7,17E-07 | -2,75 |
| ***Stfa3*** | stefin A3 | intracellular | cysteine-type endopeptidase inhibitor activity | 3,87E-10 | -2,79 |
| ***Gm10396*** | predicted gene 10396 | not determined | not determined | 4,71E-07 | -2,80 |
| ***Myocd*** | myocardin | nucleus | transcription coactivator activity | 2,06E-07 | -2,83 |
| ***Tlx1*** | T-cell leukemia, homeobox 1 | nucleus | transcription factor activity | 6,05E-08 | -2,84 |
| ***Sel1l3*** | sel-1 suppressor of lin-12-like 3 (C. elegans) | not determined | not determined // binding | 1,57E-07 | -2,87 |
| ***Krt6b*** | keratin 6B | intermediate filament | structural molecule activity | 9,61E-08 | -2,94 |
| ***Krt10*** | keratin 10 | intermediate filament | protein binding | 1,31E-05 | -3,07 |
| ***Sst*** | somatostatin | extracellular region | hormone activity | 6,01E-09 | -3,13 |
| ***Cnnm1*** | cyclin M1 | plasma membrane | not determined | 4,40E-06 | -3,19 |
| ***Serpinb12*** | serine (or cysteine) peptidase inhibitor, clade B (ovalbumin), member 12 | not determined | peptidase inhibitor activity | 7,46E-09 | -3,20 |
| ***Bmp5*** | bone morphogenetic protein 5 | extracellular region | protein binding | 3,00E-08 | -3,20 |
| ***Mcpt4*** | mast cell protease 4 | intracellular | serine-type endopeptidase activity | 6,99E-08 | -3,24 |
| ***Rxfp1*** | relaxin/insulin-like family peptide receptor 1 | plasma membrane | receptor activity | 2,96E-10 | -3,29 |
| ***Dsc1*** | desmocollin 1 | plasma membrane | protein binding | 3,23E-10 | -3,31 |
| ***Gm10001*** | predicted gene 10001 | not determined | not determined | 4,35E-10 | -3,46 |
| ***Prg4*** | proteoglycan 4 (megakaryocyte stimulating factor, articular superficial zone protein) | extracellular region | polysaccharide binding | 4,20E-08 | -3,50 |
| ***Serpinb3a*** | serine (or cysteine) peptidase inhibitor, clade B (ovalbumin), member 3A | not determined | not determined | 3,49E-07 | -3,62 |
| ***Rgs7*** | regulator of G protein signaling 7 | nucleus | GTPase activator activity | 2,90E-13 | -3,64 |
| ***Dpep1*** | dipeptidase 1 (renal) | plasma membrane | metalloexopeptidase activity | 9,65E-06 | -3,73 |
| ***Fcrl6*** | Fc receptor-like 6 | membrane | --- | 2,38E-12 | -3,96 |
| ***Cyp26c1*** | cytochrome P450, family 26, subfamily c, polypeptide 1 | --- | oxidoreductase activity | 3,10E-09 | -4,05 |
| ***Nts*** | neurotensin | extracellular region | neuropeptide hormone activity | 9,56E-10 | -4,43 |
| ***Sprr3*** | small proline-rich protein 3 | cytoplasm | protein binding | 4,79E-06 | -4,44 |
| ***4930578G10Rik*** | RIKEN cDNA 4930578G10 gene | not determined | not determined | 1,26E-06 | -5,05 |
| ***Irx4*** | Iroquois related homeobox 4 (Drosophila) | nucleus | transcription factor activity | 1,92E-07 | -5,31 |
| ***Cacna2d3*** | calcium channel, voltage-dependent, alpha2/delta subunit 3 | membrane | voltage-gated ion channel activity | 2,55E-08 | -5,66 |
| ***Mcpt2*** | mast cell protease 2 | intracellular | serine-type endopeptidase activity | 2,90E-08 | -6,39 |
| ***Isl1*** | ISL1 transcription factor, LIM/homeodomain | intracellular | chromatin binding | 3,53E-08 | -6,44 |
| ***Alx3*** | aristaless-like homeobox 3 | nucleus | transcription factor activity | 6,59E-12 | -8,36 |
| ***C130021O09Rik*** | RIKEN cDNA C130021O09 gene | not determined | not determined | 4,27E-09 | -9,31 |
| ***Pax3*** | paired box gene 3 | nucleus | chromatin binding | 1,68E-13 | -11,72 |
| ***Sfrp4*** | secreted frizzled-related protein 4 | extracellular region | Wnt-protein binding | 9,88E-10 | -12,38 |
| ***Hand2*** | heart and neural crest derivatives expressed transcript 2 | nucleus | transcription factor activity | 6,89E-14 | -13,20 |
| ***Alx1*** | ALX homeobox 1 | nucleus | transcription factor activity | 1,35E-10 | -15,95 |
| ***Hpse2*** | heparanase 2 | not determined | not determined | 2,23E-11 | -27,41 |
