# Supplementary material for: Molars and incisors: show your microarray IDs
Source: BMC Res Notes. 2013 Mar 26;6:113. doi: 10.1186/1756-0500-6-113 (PMC3658942; doi:10.1186/1756-0500-6-113)
Supplement: Additional file 4 — Overview of genes showing differential expression in developing mandibular (lower) versus maxillary (upper) molars. Only the genes exhibiting at least a two fold change in expression according to Affymetrix microarray analysis are listed. Genes with the highest expression in upper molars (positive values) or lower molars (negative values) appear on top and bottom of the list, respectively. [file 1756-0500-6-113-S4.docx]

**Additional File 4**

| **Gene Symbol** | **Gene Description** | **GO Cellular Component Term** | **GO Molecular Function Term** | **p-value** | **Fold change** |
| --- | --- | --- | --- | --- | --- |
| ***Nefl*** | neurofilament, light polypeptide | intermediate filament | structural molecule activity | 1,68E-08 | 6,28 |
| ***Ostn*** | osteocrin | extracellular region | hormone activity | 6,22E-06 | 4,12 |
| ***Nkx2-3*** | NK2 transcription factor related, locus 3 (Drosophila) | Nucleus | transcription factor activity | 5,32E-10 | 3,97 |
| ***Tnnt1*** | troponin T1, skeletal, slow | troponin complex | protein binding | 9,08E-08 | 3,19 |
| ***Chrna1*** | cholinergic receptor, nicotinic, alpha polypeptide 1 (muscle) | plasma membrane | nicotinic acetylcholine-activated cation-selective channel activity | 4,47E-06 | 3,04 |
| ***Nefm*** | neurofilament, medium polypeptide | cytoskeleton | structural molecule activity | 1,54E-08 | 3,01 |
| ***Myf5*** | myogenic factor 5 | Nucleus | transcription regulator activity | 7,14E-09 | 2,94 |
| ***Klhl31*** | kelch-like 31 (Drosophila) | not determined | protein binding | 4,58E-08 | 2,92 |
| ***Plac8*** | placenta-specific 8 | not determined | not determined | 4,68E-09 | 2,91 |
| ***Synpo2l*** | synaptopodin 2-like | cellular_componen | actin binding | 3,71E-08 | 2,87 |
| ***Slc25a21*** | solute carrier family 25 (mitochondrial oxodicarboxylate carrier), member 21 | mitochondrion | not determined | 3,15E-07 | 2,83 |
| ***Trim55*** | tripartite motif-containing 55 |  | metal ion binding | 1,29E-06 | 2,77 |
| ***Chrnd*** | cholinergic receptor, nicotinic, delta polypeptide | plasma membrane | receptor activity | 3,16E-08 | 2,77 |
| ***2510003E04Rik*** | RIKEN cDNA 2510003E04 gene | not determined | not determined | 3,60E-13 | 2,76 |
| ***Dlx6*** | distal-less homeobox 6 | Nucleus | transcription factor activity | 1,01E-07 | 2,76 |
| ***Aass*** | aminoadipate-semialdehyde synthase | mitochondrion | catalytic activity | 6,86E-09 | 2,74 |
| ***Fgfr4*** | fibroblast growth factor receptor 4 | membrane | nucleotide binding | 5,80E-08 | 2,58 |
| ***Gjb2*** | gap junction protein, beta 2 | plasma membrane | protein binding | 2,08E-08 | 2,53 |
| ***Clrn1*** | clarin 1 | membrane |  | 2,58E-08 | 2,51 |
| ***Atp1b4*** | ATPase, (Na+)/K+ transporting, beta 4 polypeptide | chromatin | sodium:potassium-exchanging ATPase activity | 5,19E-06 | 2,50 |
| ***Cdh15*** | cadherin 15 | plasma membrane | calcium ion binding | 1,88E-06 | 2,49 |
| ***Myom2*** | myomesin 2 | cytoskeleton | structural constituent of cytoskeleton | 4,88E-06 | 2,48 |
| ***Cftr*** | cystic fibrosis transmembrane conductance regulator homolog | cytoplasm | ion channel activity | 1,59E-07 | 2,46 |
| ***Gm10000*** | predicted gene 10000 | not determined | not determined | 5,61E-10 | 2,39 |
| ***Adamtsl3*** | ADAMTS-like 3 | not determined | not determined | 5,43E-07 | 2,38 |
| ***Gm9558*** | predicted gene 9558 |  |  | 2,15E-08 | 2,35 |
| ***Mypn*** | myopalladin | Nucleus | actin binding | 5,55E-06 | 2,35 |
| ***1700055N04Rik*** | RIKEN cDNA 1700055N04 gene | not determined | not determined | 8,33E-07 | 2,32 |
| ***Aldh3b2*** | aldehyde dehydrogenase 3 family, member B2 | not determined | not determined | 1,71E-06 | 2,31 |
| ***Ppfia2*** | protein tyrosine phosphatase, receptor type, f polypeptide (PTPRF), interacting protein (liprin), alpha 2 | synaptosome | protein binding | 9,74E-09 | 2,28 |
| ***Abca8a*** | ATP-binding cassette, sub-family A (ABC1), member 8a | plasma membrane | nucleotide binding | 5,45E-07 | 2,28 |
| ***Tmem45b*** | transmembrane protein 45b | integral to membrane | not determined | 4,94E-07 | 2,26 |
| ***Angptl1*** | angiopoietin-like 1 | extracellular region | receptor binding | 7,27E-07 | 2,26 |
| ***Arpp21*** | cyclic AMP-regulated phosphoprotein, 21 | cytoplasm | nucleic acid binding | 5,55E-06 | 2,26 |
| ***Krtap5-4*** | keratin associated protein 5-4 | keratin filament | not determined | 4,03E-08 | 2,23 |
| ***Fam19a1*** | family with sequence similarity 19, member A1 | extracellular region | not determined | 3,11E-06 | 2,21 |
| ***Gsc*** | goosecoid homeobox | transcription factor complex | transcription factor activity | 1,31E-06 | 2,21 |
| ***Cox8c*** | cytochrome c oxidase, subunit VIIIc | mitochondrion | cytochrome-c oxidase activity | 8,81E-09 | 2,19 |
| ***3110018I06Rik*** | RIKEN cDNA 3110018I06 gene | not determined | not determined | 5,59E-09 | 2,19 |
| ***Tceal6*** | transcription elongation factor A (SII)-like 6 | not determined | translation elongation factor activity | 2,24E-08 | 2,19 |
| ***Pitx1*** | paired-like homeodomain transcription factor 1 | Nucleus | transcription regulator activity | 6,09E-08 | 2,19 |
| ***Fitm1*** | fat storage-inducing transmembrane protein 1 | endoplasmic reticulum |  | 7,07E-07 | 2,18 |
| ***Mmd2*** | monocyte to macrophage differentiation-associated 2 | membrane | receptor activity | 2,35E-09 | 2,18 |
| ***Gm7849*** | predicted gene 7849 | not determined | not determined | 3,90E-08 | 2,18 |
| ***Arhgap36*** | Rho GTPase activating protein 36 | not determined | not determined | 1,53E-09 | 2,18 |
| ***Il1rl1*** | interleukin 1 receptor-like 1 | extracellular region | interleukin-33 receptor activity | 2,38E-06 | 2,18 |
| ***Gm9276*** | eukaryotic translation elongation factor 1 gamma pseudogene |  |  | 1,72E-08 | 2,18 |
| ***Akap14*** | A kinase (PRKA) anchor protein 14 | not determined | protein domain specific binding | 7,37E-09 | 2,17 |
| ***01-mars*** | membrane-associated ring finger (C3HC4) 1 | membrane | ligase activity | 6,38E-11 | 2,16 |
| ***Tbx4*** | T-box 4 | Nucleus | DNA binding | 3,20E-09 | 2,16 |
| ***Mug1*** | murinoglobulin 1 | extracellular region | endopeptidase inhibitor activity | 1,47E-11 | 2,16 |
| ***Fam150b*** | family with sequence similarity 150, member B | not determined | not determined | 4,80E-08 | 2,16 |
| ***Prkcq*** | protein kinase C, theta | immunological synapse | protein kinase activity | 6,71E-07 | 2,15 |
| ***Vwc2*** | von Willebrand factor C domain containing 2 | extracellular region | not determined | 4,30E-08 | 2,15 |
| ***Speer4d*** | spermatogenesis associated glutamate (E)-rich protein 4d | Nucleus | not determined | 6,33E-09 | 2,14 |
| ***Gm9717*** | predicted gene 9717 | not determined | not determined | 5,19E-07 | 2,13 |
| ***Speer4d*** | spermatogenesis associated glutamate (E)-rich protein 4d | Nucleus | not determined | 3,70E-09 | 2,12 |
| ***Olfr29-ps1*** | olfactory receptor 29, pseudogene 1 |  |  | 1,51E-09 | 2,12 |
| ***Fam26d*** | family with sequence similarity 26, member D | not determined | not determined | 3,66E-09 | 2,12 |
| ***Dok6*** | docking protein 6 | not determined | insulin receptor binding | 7,47E-08 | 2,11 |
| ***Gm8985*** | predicted gene 8985 |  |  | 1,68E-08 | 2,11 |
| ***Fibin*** | fin bud initiation factor homolog (zebrafish) | not determined | not determined | 3,53E-09 | 2,10 |
| ***Itm2a*** | integral membrane protein 2A | membrane |  | 1,36E-06 | 2,08 |
| ***A430089I19Rik*** | RIKEN cDNA A430089I19 gene | not determined | not determined | 2,41E-10 | 2,07 |
| ***1700031M16Rik*** | RIKEN cDNA 1700031M16 gene | not determined | not determined | 2,00E-08 | 2,06 |
| ***Olfr444*** | olfactory receptor 444 | integral to membrane | G-protein coupled receptor activity | 1,01E-06 | 2,05 |
| ***AI593442*** | expressed sequence AI593442 | not determined | not determined | 2,08E-06 | 2,05 |
| ***Gm5734*** | predicted gene 5734 | not determined | not determined | 5,93E-08 | 2,03 |
| ***B3galt2*** | UDP-Gal:betaGlcNAc beta 1,3-galactosyltransferase, polypeptide 2 | Golgi apparatus | galactosyltransferase activity | 1,91E-06 | 2,03 |
| ***Grap2*** | GRB2-related adaptor protein 2 |  | protein binding | 1,12E-07 | 2,02 |
| ***Ankrd1*** | ankyrin repeat domain 1 (cardiac muscle) | transcription factor complex | transcription corepressor activity | 5,84E-07 | 2,01 |
| ***Chst7*** | carbohydrate (N-acetylglucosamino) sulfotransferase 7 | Golgi membrane | sulfotransferase activity | 2,24E-08 | 2,01 |
| ***9130404H23Rik*** | RIKEN cDNA 9130404H23 gene | not determined | not determined | 8,26E-09 | 2,01 |
| ***Ccl26*** | chemokine (C-C motif) ligand 26 | extracellular space | cytokine activity | 2,61E-10 | 2,01 |
| ***Corin*** | corin | plasma membrane | serine-type endopeptidase activity | 4,37E-09 | 2,01 |
| ***Barx2*** | BarH-like homeobox 2 | Nucleus | chromatin binding | 3,47E-07 | 2,01 |
| ***Otx1*** | orthodenticle homolog 1 (Drosophila) | Nucleus | transcription factor activity | 8,02E-08 | 2,00 |
| ***Llph*** | LLP homolog, long-term synaptic facilitation (Aplysia) | not determined | not determined | 5,36E-07 | -2,01 |
| ***Mdga2*** | MAM domain containing glycosylphosphatidylinositol anchor 2 | plasma membrane |  | 3,76E-06 | -2,02 |
| ***Gabra1*** | gamma-aminobutyric acid (GABA) A receptor, subunit alpha 1 | plasma membrane | GABA-A receptor activity | 1,63E-08 | -2,05 |
| ***Rorb*** | RAR-related orphan receptor beta | Nucleus | transcription factor activity | 6,41E-09 | -2,09 |
| ***Zmat4*** | zinc finger, matrin type 4 | not determined | nucleic acid binding | 2,84E-07 | -2,14 |
| ***Fcrl6*** | Fc receptor-like 6 | membrane |  | 6,03E-11 | -2,17 |
| ***Rtn4rl1*** | reticulon 4 receptor-like 1 | plasma membrane | receptor activity | 4,35E-07 | -2,20 |
| ***Naalad2*** | N-acetylated alpha-linked acidic dipeptidase 2 | not determined | carboxypeptidase activity | 6,48E-09 | -2,21 |
| ***Kcnb2*** | potassium voltage gated channel, Shab-related subfamily, member 2 | voltage-gated potassium channel complex | voltage-gated ion channel activity | 1,42E-06 | -2,30 |
| ***Itga8*** | integrin alpha 8 | integrin complex | receptor activity | 4,57E-08 | -2,34 |
| ***Gabrb2*** | gamma-aminobutyric acid (GABA) A receptor, subunit beta 2 | membrane fraction | ion channel activity | 1,21E-07 | -2,60 |
| ***ATP6*** | ATP synthase F0 subunit 6 | mitochondrion | hydrogen ion transmembrane transporter activity | 1,26E-07 | -2,84 |
| ***Alx1*** | ALX homeobox 1 | Nucleus | transcription factor activity | 2,50E-08 | -2,85 |
| ***2610017I09Rik*** | RIKEN cDNA 2610017I09 gene | not determined | not determined | 1,52E-10 | -2,92 |
| ***Gabrb2*** | gamma-aminobutyric acid (GABA) A receptor, subunit beta 2 | cell junction | ion channel activity | 7,32E-09 | -3,06 |
| ***Ndst4*** | N-deacetylase/N-sulfotransferase (heparin glucosaminyl) 4 | Golgi apparatus | catalytic activity | 4,21E-06 | -3,48 |
| ***Pla2g7*** | phospholipase A2, group VII (platelet-activating factor acetylhydrolase, plasma) | extracellular region | hydrolase activity | 1,34E-08 | -3,99 |
| ***Nmbr*** | neuromedin B receptor | plasma membrane | receptor activity | 5,52E-10 | -4,25 |
| ***Cyp26c1*** | cytochrome P450, family 26, subfamily c, polypeptide 1 |  | monooxygenase activity | 1,74E-10 | -5,04 |
