# Supplementary material for: Molars and incisors: show your microarray IDs
Source: BMC Res Notes. 2013 Mar 26;6:113. doi: 10.1186/1756-0500-6-113 (PMC3658942; doi:10.1186/1756-0500-6-113)
Supplement: Additional file 6 — Sequences of primers used for real-time qRT-PCR assays. [file 1756-0500-6-113-S6.docx]

**Additional File 6**

| **Genes/Primers** | **Sequences (5' to 3')** |
| --- | --- |
| Adamtsl3 For. | CCTACAATGACGTGCAGTACC |
| Adamtsl3 Rev. | GTTCCATCCAACACTTTGGGT |
| Alx1 For. | CCTTCACACCGAGCTGAATAG |
| Alx1 Rev. | GACATCCTGAGATTGTTGCAGT |
| Bmp5 For. | TTACTTAGGGGTATTGTGGGCT |
| Bmp5 Rev. | TGAACGTGATTGTCTCCCAAG |
| Cyp1b1 For. | CTTCGCCTCTTTCCGTGTG |
| Cyp1b1 Rev. | GTGACCGAACGCCAGACTG |
| Cyp26c1 For. | TGGCCCAACAACTCTGGAC |
| Cyp26c1 Rev. | CAGCGTTTCACCGAAGAATGG |
| Dll1 For. | CCCATCCGATTCCCCTTCG |
| Dll1 Rev. | GGTTTTCTGTTGCGAGGTCATC |
| Fgf12 For. | CTAATTCCTGTAGGACTGCGTG |
| Fgf12 Rev. | GGGGTAAAAACATCTGAGCTGT |
| Fgfr4 For. | TACACATGCCTTGTGGAGAAC |
| Fgfr4 Rev. | GGAGATAGCTGTAGCGAATGC |
| Gapdh For. | AGGTCGGTGTGAACGGATTTG |
| Gapdh Rev. | TGTAGACCATGTAGTTGAGGTCA |
| Gl1 Rev. | CTTCCGAGTCAGACAGTCCCT |
| Gli1 For. | TACCATGAGCCCTTCTTTAGGA |
| Ihh For. | CTCAGACCGTGACCGAAATAAG |
| Ihh Rev. | CCTTGGACTCGTAATACACCCAG |
| Itga8 For. | TGTCTGGCGTTCAACTTGGAT |
| Itga8 Rev. | TCCAGTGAGTAGCCGAAGTAG |
| Prkcq For. | GAGATGCCGCAAGAACAATGG |
| Prkcq Rev. | ATTCATTAGCATTCGGCCTTGA |
| Rorb For. | AGGAACCGTTGCCAACACTG |
| Rorb Rev. | GACATCCTCCCGAACTTTACAG |
| Sfrp1 For. | TACTGGCCCGAGATGCTCAA |
| Sfrp1 Rev. | GAGGCTTCCGTGGTATTGGG |
| Sfrp2 For. | GGCCACGAGACCATGAAGG |
| Sfrp2 Rev. | CATGACCAGCGGAATCCAGG |
| Shox2 For. | CAAAGACGATGCGAAAGGGAT |
| Shox2 Rev. | AGGGTAAAATTGGTCCGACTTC |
| Smoc2 For. | GAAGGAGTTCCAGCAAGTGTT |
| Smoc2 Rev. | AGTATCCTGTGTAGCTGTGACA |
| Wnt11 For. | AAACTGATGCGTCTACACAA |
| Wnt11Rev. | CATGGCATTTACACTTCGTTTCC |
